# Is mindfulness research methodology improving over time? A systematic review

**DOI:** 10.1371/journal.pone.0187298

**Published:** 2017-10-31

**Authors:** Simon B. Goldberg, Raymond P. Tucker, Preston A. Greene, Tracy L. Simpson, David J. Kearney, Richard J. Davidson

**Affiliations:** 1 VA Puget Sound Health Care System–Seattle Division, Seattle, Washington, United States of America; 2 Center for Healthy Minds, University of Wisconsin—Madison, Madison, WI, United States of America; 3 Department of Counseling Psychology, University of Wisconsin—Madison, Madison, WI, United States of America; 4 Department of Psychology, Louisiana State University, Baton Rouge, LA, United States of America; 5 Center for Excellence in Substance Abuse Treatment & Education, VA Puget Sound Health Care System–Seattle Division, Seattle, Washington, United States of America; 6 Department of Psychology, University of Wisconsin—Madison, Madison, WI, United States of America; University of Groningen, NETHERLANDS

## Abstract

**Background:**

Despite an exponential growth in research on mindfulness-based interventions, the body of scientific evidence supporting these treatments has been criticized for being of poor methodological quality.

**Objectives:**

The current systematic review examined the extent to which mindfulness research demonstrated increased rigor over the past 16 years regarding six methodological features that have been highlighted as areas for improvement. These feature included using active control conditions, larger sample sizes, longer follow-up assessment, treatment fidelity assessment, and reporting of instructor training and intent-to-treat (ITT) analyses.

**Data sources:**

We searched PubMed, PsychInfo, Scopus, and Web of Science in addition to a publically available repository of mindfulness studies.

**Study eligibility criteria:**

Randomized clinical trials of mindfulness-based interventions for samples with a clinical disorder or elevated symptoms of a clinical disorder listed on the American Psychological Association’s list of disorders with recognized evidence-based treatment.

**Study appraisal and synthesis methods:**

Independent raters screened 9,067 titles and abstracts, with 303 full text reviews. Of these, 171 were included, representing 142 non-overlapping samples.

**Results:**

Across the 142 studies published between 2000 and 2016, there was no evidence for increases in any study quality indicator, although changes were generally in the direction of improved quality. When restricting the sample to those conducted in Europe and North America (continents with the longest history of scientific research in this area), an increase in reporting of ITT analyses was found. When excluding an early, high-quality study, improvements were seen in sample size, treatment fidelity assessment, and reporting of ITT analyses.

**Conclusions and implications of key findings:**

Taken together, the findings suggest modest adoption of the recommendations for methodological improvement voiced repeatedly in the literature. Possible explanations for this and implications for interpreting this body of research and conducting future studies are discussed.

## Introduction

The past several decades have seen a remarkable increase in scientific interest in mindfulness-based interventions. Beginning with mindfulness-based stress reduction (MBSR) [1] which was based largely on Buddhist contemplative practices [2], numerous mindfulness-based interventions that target a range of psychiatric and medical conditions have been developed and tested (e.g., mindfulness-based cognitive therapy for depression [MBCT], mindfulness-based eating awareness training [MB-EAT] for binge eating, mindfulness training for smokers [MTS] for smoking cessation, mindfulness-oriented recovery enhancement [MORE] for chronic pain and opiate misuse) [3–6]. Broadly speaking, there is evidence that these interventions show beneficial effects for both adults and children and across a variety of outcomes [7–9].

Despite promising effects demonstrated in meta-analyses of randomized clinical trials (RCTs), concerns have continually been raised regarding the methodological quality of this body of research. Bishop (2002) [10] offered some of the earliest criticisms of the research methods employed in studies on mindfulness, noting that claims of efficacy may be overstated. Bishop noted that although MBSR was being widely used clinically for the management of stress associated with chronic illness as well as for treating some psychiatric conditions, the published literature was “rife with methodological problems” (p. 71) [10]. Bishop highlights a host of concerns including a lack of active comparison groups that allow evaluation of the impact of non-specific benefits (e.g., social support) and controlling for time and attention, a relative absence of follow-up assessment of outcomes, and a lack of measurement of specific threats to validity due to response set biases (e.g., social desirability).

Many of these concerns were also raised in one of the first meta-analyses in this area. Baer (2003) [11] included 21 studies in a meta-analysis, reporting the effects of mindfulness-based interventions on chronic pain, Axis I disorders, other mental health disorders, mixed clinical populations, and nonclinical populations, with an overall mean effect weighted by sample size of *d* = 0.59. Like Bishop (2002) [10], Baer (2003) highlighted the limitation of studies that lacked a control condition (i.e., pre-post designs) as well as studies relying on non-active control conditions. Baer (2003) noted that although a treatment-as-usual control will account for change due to the passage of time, an active control is needed to account for the influence of demand characteristics and placebo effects (i.e., non-specific factors). Both Bishop and Baer note the importance of comparing mindfulness interventions to other therapies (e.g., cognitive behavioral therapy). Baer also discussed limitations related to the small size of samples in many of the trials and a lack of evaluation of treatment integrity (i.e., discussion of training and supervision of therapists, assessment of treatment fidelity and adherence).

Over a decade and hundreds of RCTs later, researchers continue to offer strikingly similar critiques of the mindfulness literature. Davidson and Kaszniak (2015) [12] emphasize the impossibility of the double-blind placebo-controlled design in mindfulness research and echo calls for both plausible and therapeutic comparison conditions (e.g., health enhancement program [HEP]) [13]. The authors call for more consistent reporting and evaluation of treatment fidelity, instructor training, and instructor credibility along with the inclusion of intent-to-treat (ITT) analyses. Kuyken and colleagues (2016) [14] also stressed the importance of assessing the relative advantage of MBCT through comparisons with active control conditions, the reporting of treatment fidelity, and the need for longer follow-up assessment. It is precisely these methodological shortcomings (along with others, such as selective reporting biases) [15] that continue to raise questions regarding the evidence base for mindfulness interventions [16].

Have researchers taken up these suggestions? In the years since Bishop (2002) [10] and Baer (2003) [11], is there evidence that the methodological rigor of mindfulness research has improved? The current systematic review sought to address this question empirically. In particular, we examined six methodological features that have been recommended in criticisms of mindfulness research [10–12. 14]. These include: (a) active control conditions, (b) larger sample sizes, (c) longer follow-up assessment, (d) treatment fidelity assessment, (e) reporting of instructor training, (f) reporting of ITT samples.

It is worth briefly describing these six features and their importance. As described below, we graded the strength of the control condition on a five-tier system. We defined specific active control conditions as comparison groups that were intended to be therapeutic [17]. More rigorous control groups are important as they can provide a test of the unique or added benefit a mindfulness intervention may offer, beyond non-specific benefits associated with the placebo effect, researcher attention, or demand characteristics [11,14]. Larger sample sizes are important as they increase the reliability of reported effects and increase statistical power [11]. Longer follow-up is important for assessing the degree to which treatment effects are maintained beyond the completion of the intervention [10]. Treatment fidelity assessment allows an examination of the degree to which the given treatment was delivered as intended [12]. Treatment fidelity is commonly assessed through video or audio recordings of sessions that are coded and/or reviewed by treatment experts [18]. We coded all references to treatment fidelity assessment (e.g., sessions were recorded and reviewed, a checklist measuring adherence to specific treatment elements was completed). Relatedly, reporting of instructor training increases the likelihood that the treatment that was delivered by qualified individuals [12], which should, in theory, influence the quality of the treatment provided. Lastly, the reporting of ITT analyses involves including individuals who may have dropped out of the study and/or did not complete their assigned intervention [12]. Generally speaking, ITT analyses are viewed to be more conservative estimates of treatment effects [19,20], and are preferred for this reason.

As there are now a large number of published RCTs in this area [7], our review focused on studies using randomized designs. We were interested in the evidence base for mindfulness as a clinical intervention, so we only included samples drawn from clinical populations. Given our interest in exploring the strength of the comparison conditions used (including comparisons with evidence-based treatments [EBTs]) and the psychosocial nature of mindfulness as an intervention, we restricted our sample to disorders listed on the American Psychological Association’s (APA) Division 12 (Society of Clinical Psychology; see Table in S1 Table) EBTs list [21]. The use of Division 12’s list also allowed assessment of the extent to which frontline EBTs are being used as comparison groups. Analyses focused on the extent to which the methodological suggestions noted above are being incorporated into the empirical literature over time.

## Method

### Eligibility criteria

We included all RCTs of mindfulness-based interventions for adult patients with psychiatric diagnoses for which there are evidence-based treatments per the American Psychological Association’s Division 12 (Society of Clinical Psychology; see Table in S1 Table). To be eligible, samples had to be comprised of participants with either a formal diagnosis or elevated symptoms of a given disorder. Studies conducted in treatment facilities focused on a specific disorder (e.g., substance abuse treatment) were included. Elevated stress levels alone were not considered to reflect a clinical condition.

To qualify, interventions had to have mindfulness meditation as a core component with home meditation practice as a treatment ingredient. While interventions combining mindfulness with other modalities (e.g., mindfulness and cognitive techniques as in Mindfulness-Based Cognitive Therapy [MBCT]) [6] were included, therapies emphasizing the attitudinal stance of mindfulness (rather than the formal practice of mindfulness meditation) were excluded (e.g., Acceptance and Commitment Therapy [ACT], Dialectical Behavior Therapy [DBT]) [22,23]. Other forms of meditation (e.g., mantram repetition) were excluded. Interventions had to be delivered in real time (i.e., not provided through video instruction) and had to include more than one session (to allow for home meditation practice). Studies were also excluded for the following reasons: (1) not published in English; (2) not a peer-reviewed article; (3) data unavailable to compute standardized effect sizes; (4) no disorder-specific (i.e., targeted) outcomes reported; (5) data redundant with other included studies; (6) no non-mindfulness-based intervention or condition included.

### Information sources

We searched the following databases: PubMed, PsycInfo, Scopus, Web of Science. In addition, a publicly available comprehensive repository of mindfulness studies was also searched [24]. Citations from recent meta-analyses and systematic reviews were also included [7,8]. Citations were included from the first available date until January 2^nd^, 2017.

### Search

We used the search terms “mindfulness” and “random*”. When a database allowed (e.g., PsycInfo), we restricted our search to clinical trials.

### Study selection

Titles and/or abstracts of potential studies were independently coded by the first author and a second co-author. Disagreements were discussed with the senior author until consensus was reached.

### Data collection process

Standardized spreadsheets were developed for coding both study-level and effect size-level data. Coders were trained by the first author through coding an initial sample of studies (*k* = 10) in order to achieve reliability. Data were extracted independently by the first author and a second co-author. Disagreements were again discussed with the senior author. Inter-rater reliabilities were in the good to excellent range (i.e., *K*s and *ICC*s > .60) [25].

### Data items

Along with data necessary for computing standardized effect sizes, the following data were extracted: (1) publication year; (2) disorder; (3) sample demographics (mean age, percentage female, percentage with some college education); (4) country of origin; (5) intent-to-treat (ITT) sample size; (6) length of longest follow-up (i.e., assessments occurring after immediately post-treatment assessment); (7) whether treatment fidelity was assessed; (8) whether the training of instructors was reported; (9) whether an ITT analysis was reported; (10) whether a non-self-report outcome was included; (11) whether the control condition matched treatment time with the mindfulness condition; (12) quality of the control condition. Quality of the control condition was assessed based on a five-tier system. These included: (1) no treatment (in which the control condition received no intervention beyond that which was provided to the treatment condition); (2) minimal treatment (e.g., instruction in management of depressive symptoms through self-monitoring questionnaires); (3) non-specific active control (active conditions in which no mechanism of change or clear rationale for treatment was provided); (4) specific active control (contained specific therapeutic mechanisms, has a theoretical / treatment rationale); (5) evidence-based treatment (EBT). Comparison treatments were coded as EBTs if they were identified by APA Division 12 as an EBT for that particular disorder, or if they were promoted as a first-line treatment by a similarly relevant organization (e.g., smoking cessation treatment promoted by the American Lung Association). When studies included multiple control conditions, the most rigorous was used in analyses on the strength of the control condition.

### Summary measures

As our study was aimed at addressing whether the methodological rigor of mindfulness research has improved over time, the six key study design features served as our dependent variables with year of publication as the independent variable. While numerous design features could have been examined (indeed, a recent review of study quality measures identified 185 different characteristics that have been recommended) [26], we focused on six features that have been repeatedly identified in the mindfulness literature as areas for improvement. Ordinary least squares (OLS) and logistic regression models were used to assess changes over time using the R statistical software [27]. To ease interpretation, standardized effect sizes were also computed (as *ß*s for OLS regression models and odd ratios [*OR*] for logistic regression models). Three sets of sensitivity analyses were run. The first involved restricting the sample to studies conducted in Europe and North America, given scientific research on secular forms of mindfulness has the longest history in these regions (e.g., MBSR, MBCT) [1,6]. The second involved excluding an early, high-quality study [28] (i.e., *N* = 145, 12 month follow-up, fidelity was assessed, instructor training was reported, ITT analysis was reported) whose year of publication was over three standard deviations below the mean and that could potentially exert high leverage in the regression models and unduly influence results. The third involved assessing the impact of log-transforming the predictor (year of publication) and response variables (ITT sample size, length of follow-up) on the normality of the residuals and model results in the OLS regression models (which assume normally distributed residuals) [29].

## Results

### Study selection

**Fig 1:**
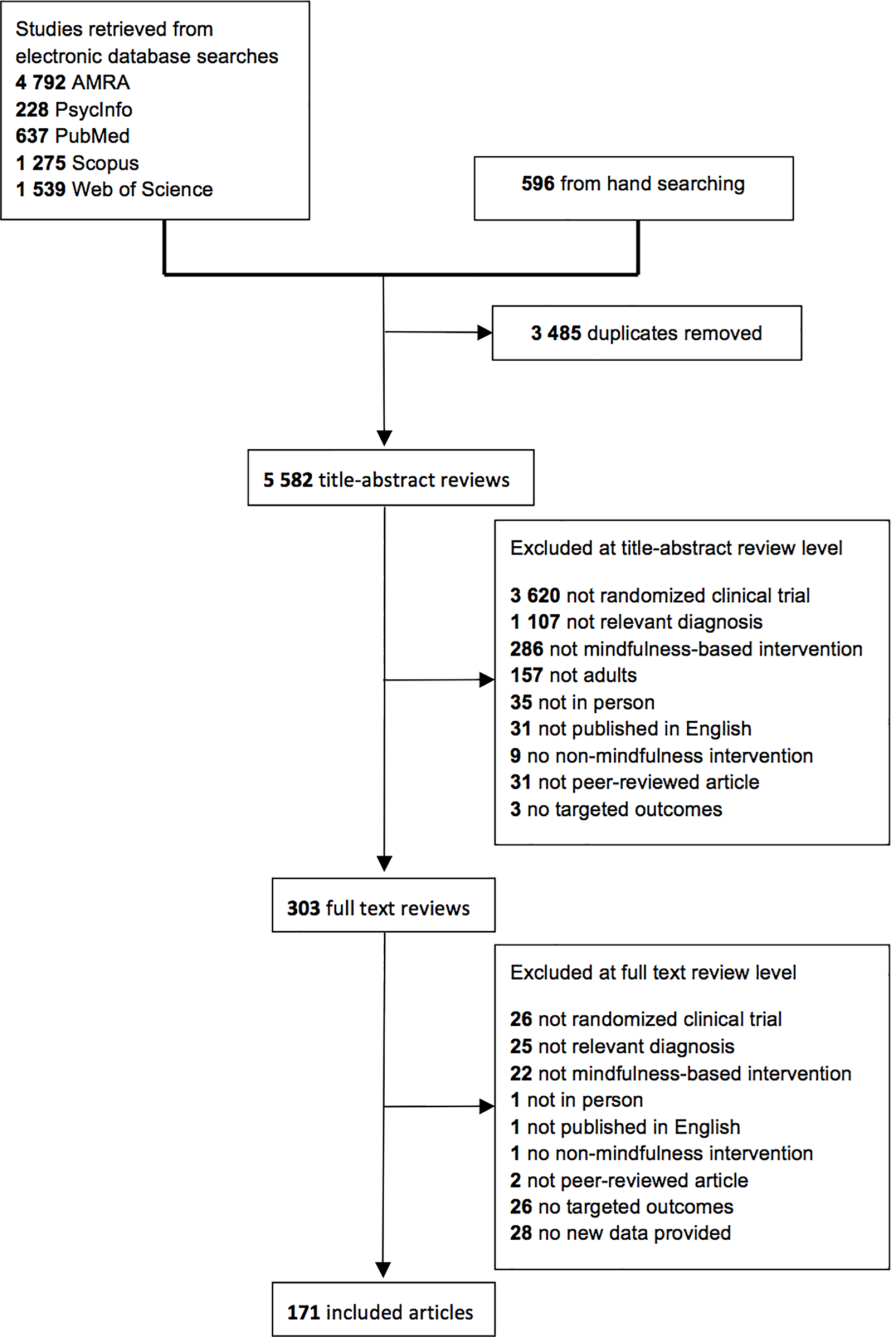
PRISMA flow diagram.

A total of 9,067 citations were retrieved. After 3,485 duplicates were removed, 5,582 unique titles and/or abstracts were coded. Following the application of the exclusion criteria (see PRISMA flow diagram; Fig 1), 171 articles representing 142 studies were retained for analysis. This sample included 164 unique comparisons (i.e., pairings between a mindfulness condition and a control condition) and 12,005 participants.

### Study characteristics

Study characteristics, including the six methodological features of interest, are reported for each study in Table in S2 Table. All studies were published between 2000 and 2016. The sample was on average 43.56 years old, 64.21% female, with 61.21% having some post-secondary education. The largest percentage of trials was conducted in the United States (44.37%). The largest proportion of studies used no treatment comparison conditions (52.44%). The most commonly studied disorder was depression (30.82%).

Descriptive statistics for the six study design features are presented in Table 1. Some features were commonly included (e.g., reporting of mindfulness instructor training) while others were less common (e.g., treatment fidelity assessment).

**Table 1:** Study quality descriptive statistics.

Characteristic	*k*	%	Mean	*SD*
Includes active control condition	72	50.70		
Includes therapeutic control condition	65	45.77		
Includes EBT control condition	27	19.01		
Sample size			84.54	71.19
Includes follow-up assessment	79	55.63		
Longest follow-up (months, all studies)			3.58	5.11
Longest follow-up (months, studies with follow-up)			6.43	5.36
Treatment fidelity assessed	46	32.39		
Instructor mindfulness training reported	104	73.24		
Protocol specific mindfulness training reported	90	63.38		
ITT analysis reported	93	65.49		

Notes: *k* = number of studies with given characteristic (out of 142 total studies); EBT = evidence-based treatment; ITT = intent-to-treat.

### Risk of bias within studies

All included studies used randomized designs. The majority of comparisons did not match treatment time between the mindfulness and control conditions (59.14%) and the majority of studies reported an ITT analysis (65.49%). Approximately half of the studies included a non-self-report measure (48.59%).

### Results of individual studies

For each included study, description of the six methodological features assessed are reported in Supplemental Materials.

### Synthesis of results

#### Strength of the comparison condition

**Fig 2:**
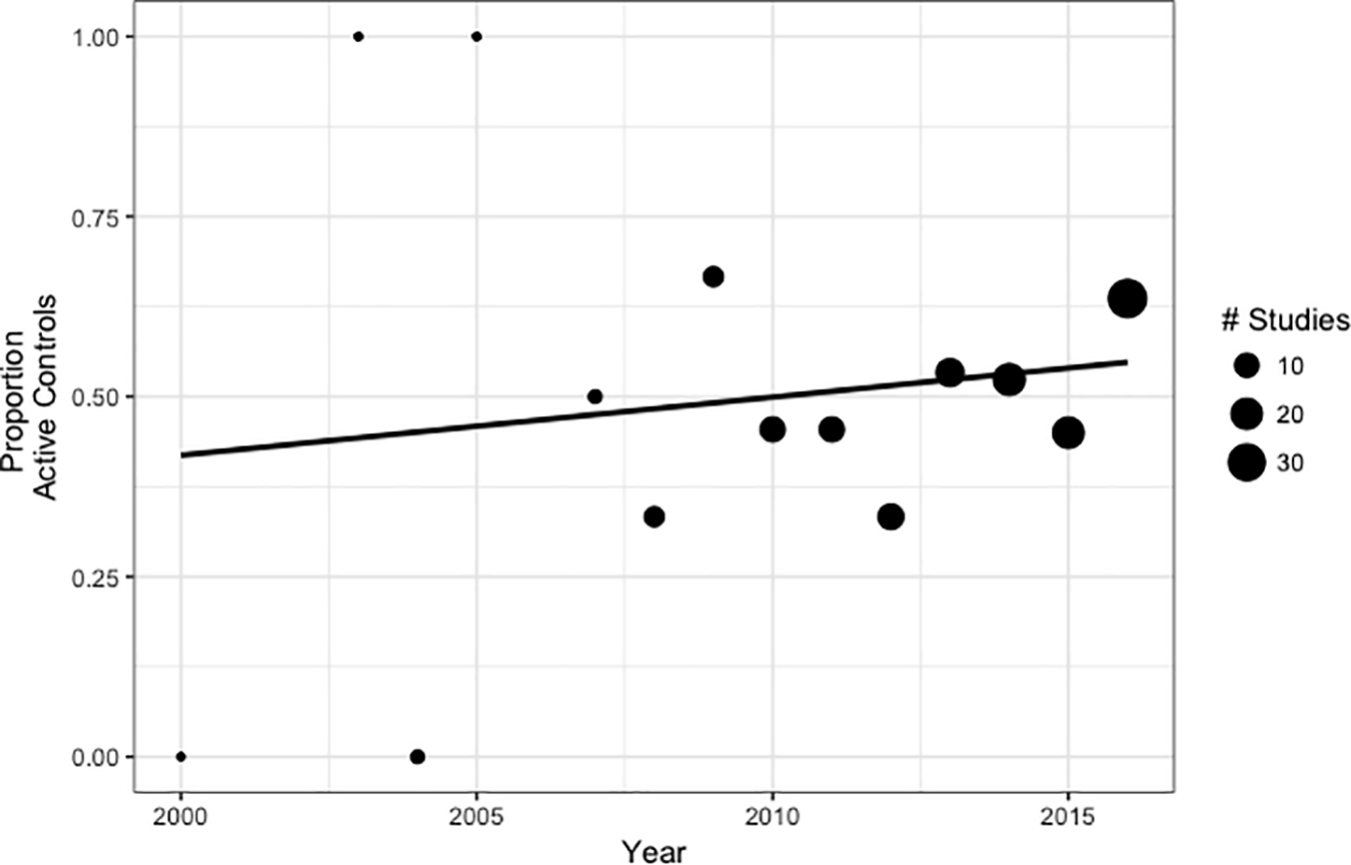
Proportion of studies using active control conditions over time. The size of each point is relative to the number of studies represented.

The first analysis examined whether the likelihood of a study including an active control condition increased over time. There was no evidence that more recent studies are more likely to include an active control condition (*B* = 0.072, *OR =* 1.07, *p* = .195, Table 2, Fig 2). Similarly, there was no evidence that comparison conditions that are intended to be therapeutic (i.e., specific active controls, EBTs) were being used more frequently over time. Results were unchanged (i.e., *p*s > .10) when the sample was restricted to studies conducted in Europe and North America or when excluding Teasdale et al. (2000) [28].

**Table 2:** Study quality predicted by year of publication (full sample).

Model	Outcome	B	*SE*	*ß / OR*	*z*- / *t*-value	*df*	*p*-value
Comparison type	Active control^a^	0.072	0.056	1.07	1.30	140	.195
	Specific active or EBT^a^	0.045	0.055	1.05	0.82	140	.414
	EBT^a^	0.00087	0.069	1.00	0.013	140	.990
Sample size	Sample size	3.35	1.92	0.15	1.75	140	.082
Follow-up	Any follow-up assessment^a^	0.048	0.055	1.05	0.88	140	.378
	Length of follow-up	-0.073	0.18	-0.05	-0.41	77	.686
	Length of follow-up (includes zero)	0.028	0.14	0.02	0.20	140	.839
Treatment fidelity	Treatment fidelity reported^a^	0.13	0.067	1.14	1.94	140	.053
Instructor training	Any training in mindfulness^a^	0.019	0.060	1.02	0.31	140	.755
	Protocol specific training^a^	0.003	0.056	1.00	0.053	140	.958
ITT analysis	ITT analysis reported^a^	0.10	0.057	1.11	1.81	140	.070

Note: B = unstandardized regression coefficient; *SE* = standard error; *ß* = standardized regression coefficient; *OR* = odds ratio; *df* = degrees of freedom; EBT = evidence-based treatment; ITT = intent-to-treat.

^a^ = logistic regression model used (and odds ratios reported).

#### Sample size

**Fig 3:**
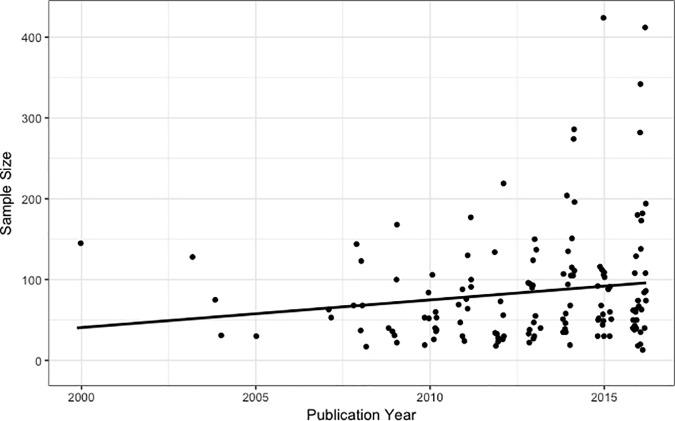
Changes in sample size over time.

The second set of analyses examined whether sample sizes have increased over time. There was a marginally significant increase in sample size over time (*B* = 3.35, *ß* = 0.15, *p* = .082, Table 2, Fig 3). This effect remained marginally significant when examining only studies conducted in Europe or North American (*B* = 4.16, *ß* = 0.18, *p* = .060, Table 3). When excluding Teasdale et al. (2000) [28], a significant increase in sample size over time was found (*B* = 4.47, *ß* = 0.18, *p* = .030; Table 4). The residuals in these models were normalized when both year of publication and sample size were log-transformed, with significance tests unchanged (*B*s = -0.13, -0.15, and -0.15, *p*s = .082, .084, and .048; for the full sample, North American and European sample, and full sample with Teasdale et al. (2000) excluded, respectively).

**Table 3:** Study quality predicted by year of publication (European and North American sample only).

Model	Outcome	B	*SE*	*ß/ OR*	*z*- / *t*-value	*df*	*p*-value
Comparison type	Active control^a^	0.086	0.059	1.09	1.45	109	.146
	Specific active or EBT^a^	0.057	0.059	1.06	0.97	109	.334
	EBT^a^	0.035	0.073	1.04	0.48	109	.635
Sample size	Sample size	4.16	2.19	0.18	1.90	109	.060
Follow-up	Any follow-up assessment^a^	0.038	0.058	1.04	0.67	109	.506
	Length of follow-up	-0.054	0.17	-0.04	-0.32	57	.750
	Length of follow-up (includes zero)	0.021	0.13	0.02	0.16	109	.872
Treatment fidelity	Treatment fidelity reported^a^	0.12	0.071	1.13	1.75	109	.081
Instructor training	Any training in mindfulness^a^	0.070	0.066	1.07	1.07	109	.283
	Protocol specific training^a^	0.040	0.06	1.04	0.66	109	.509
ITT analysis	ITT analysis reported^a^	0.12	0.061	1.13	1.98	109	.048

Note: B = unstandardized regression coefficient; *SE* = standard error; *ß =* standardized regression coefficient; *OR* = odds ratio; *df* = degrees of freedom; EBT = evidence-based treatment; ITT = intent-to-treat.

^a^ = logistic regression model used (and odds ratios reported).

**Table 4:** Study quality predicted by year of publication (Teasdale et al. (2000) excluded).

Model	Outcome	B	*SE*	*ß / OR*	*z*- / *t*-value	*df*	*p*-value
Comparison type	Active control^a^	0.059	0.058	1.06	1.01	139	.311
	Specific active or EBT^a^	0.031	0.058	1.03	0.53	139	.596
	EBT^a^	-0.012	0.073	0.99	-0.17	139	.867
Sample size	Sample size	4.47	2.03	0.18	2.20	139	.030
Follow-up	Any follow-up assessment^a^	0.075	0.059	1.08	1.27	139	.204
	Length of follow-up	0.011	0.20	0.01	0.053	76	.958
	Length of follow-up (includes zero)	0.12	0.15	0.07	0.84	139	.404
Treatment fidelity	Treatment fidelity reported^a^	0.20	0.076	1.22	2.58	139	.010
Instructor training	Any training in mindfulness^a^	0.036	0.064	1.04	0.56	139	.577
	Protocol specific training^a^	0.020	0.060	1.02	0.34	139	.734
ITT analysis	ITT analysis reported^a^	0.13	0.061	1.14	2.19	139	.029

Note: B = unstandardized regression coefficient; *SE* = standard error; *ß* = standardized regression coefficient; *OR* = odds ratio; *df* = degrees of freedom; EBT = evidence-based treatment; ITT = intent-to-treat.

^a^ = logistic regression model used (and odds ratios reported).

#### Length of follow-up

**Fig 4:**
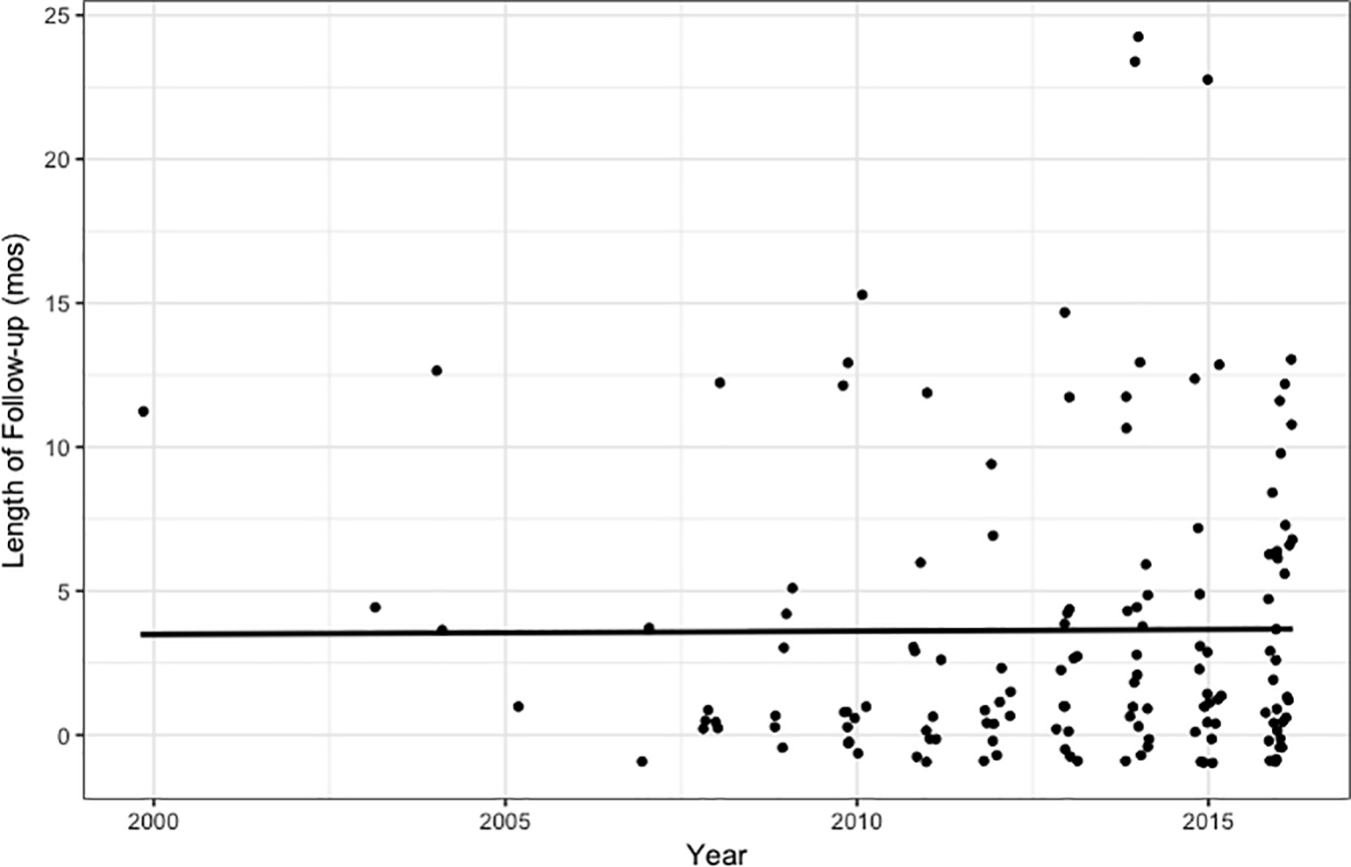
Changes in length of follow-up over time.

The third set of analyses assessed follow-up data collection as well as the length of the longest follow-up. There was no increase in likelihood that a given study would include follow-up assessments (*B* = 0.048, *OR* = 1.05, *p* = .378). For studies that included a follow-up time point, there was no evidence that length of follow-up has increased over time (*B* = -0.073, *ß* = -0.05, *p* = .686). Results were unchanged (i.e., *p*s > .10) when the sample was restricted to studies conducted in Europe and North America, when excluding Teasdale et al. (2000) [28], or when coding studies without follow-up as having a follow-up length of zero (Fig 4). The residuals in these models were normalized when both year of publication and length of follow-up were log-transformed, with significance tests unchanged (length of follow-up: *B*s = 0.07, 0.00, and 0.04, *p*s = .572, .994, and .769; for the full sample, North American and European sample, and full sample with Teasdale et al. (2000) excluded, respectively; length of follow-up including zeros: *B*s = 0.02, 0.03, and 0.03; *p*s = .688, .385, and .563; for the full sample, North American and European sample, and full sample with Teasdale et al. (2000) excluded, respectively).

#### Reporting of fidelity assessment

The fourth set of analyses examined whether treatment fidelity was assessed and reported. Less than half of the studies (32.39%) assessed and reported treatment fidelity. A marginally significant increase in the reporting of treatment fidelity was seen in the full sample (*B* = 0.13, *OR =* 1.14, *p* = .053). This effect remained marginally significant when examined in studies conducted in Europe and North American (*B* = 0.12, *OR* = 1.13, *p* = .081). When excluding Teasdale et al. (2000) [28], a significant increase in the reporting of fidelity assessment was detected over time (*B* = 0.20, *OR =* 1.22, *p* = .010).

#### Reporting instructor training

The fifth set of analyses examined whether studies reported instructors receiving specialized training in the mindfulness protocol being delivered. The majority of studies reported that instructors had prior training in mindfulness (73.23%), with a majority also reporting training related to the specific mindfulness protocol being delivered (63.38%). There was no evidence that more recent studies were more likely to report either prior training in mindfulness (*B* = 0.019, *OR* = 1.02, *p* = .755) or training in the specific mindfulness protocol being delivered (*B* = 0.0030, *OR* = 1.00, *p* = .958). Results were unchanged (i.e., *p*s > .10) when the sample was restricted to studies conducted in Europe and North America or when excluding Teasdale et al. (2000) [28].

#### Reporting of intent-to-treat analyses

The sixth set of analyses examined whether studies reported an intent-to-treat (ITT) analysis. A marginally significant increase in this practice was evident in the full sample (*B* = 0.10, *OR* = 1.11, *p* = .070). This effect was significant when examined in the European and North American portions of the sample (*B* = 0.12, *OR* = 1.13, *p* = .048) and when excluding Teasdale et al. (2000; *B* = 0.13, *OR* = 1.14, *p* = .029) [28].

## Discussion

The current systematic review aimed to assess the degree to which mindfulness research has improved methodologically over time. We examined six study design features that have repeatedly been suggested as areas for improvement in reviews of the literature [11,12]. On the whole, there was only modest evidence that the quality of mindfulness research has improved. Of the six study design features assessed in this review, no significant increases were noted in the full sample, and effect sizes were very small based on standard guidelines [30,31]. Marginally significant increases were seen in sample size, treatment fidelity assessment, and the reporting of ITT analyses. In RCTs conducted in Europe and North America, a significant increase in the reporting of ITT analyses was found, along with marginally significant increases in sample size and treatment fidelity assessment. When an early, high-quality study was excluded [28], whose year of publication was over three standard deviations below the mean, improvements were seen in sample size, treatment fidelity assessment, and ITT analysis reporting. For the design feature most often emphasized in criticisms of mindfulness research–the lack of active control conditions–no increases were detected, and the odds ratios reflecting this effect size were quite small (*OR*s = 1.06 to 1.09). This is unfortunate, given comparisons with other active therapies are essential for addressing the relative efficacy of mindfulness treatments. Similarly, there was no evidence that newer studies are more likely to include follow-up assessment (*OR*s = 1.04 to 1.08), a second key design feature of establishing the efficacy of mindfulness interventions.

In some ways, these results are discouraging. Considerable scientific efforts (and financial resources) have been spent conducting research on mindfulness (see the exponential growth of publications in this area) [32], yet the body of literature is, on average, not becoming more rigorous with time. This fact suggests Bishop’s (2002) critique of this area as “rife with methodological problems” (p. 71) [10] remains valid over a decade later.

At once, several other perspectives are worth considering. One is that, due to the increased publication in this area, the accumulation of high-quality studies is occurring nonetheless, even if these studies do not represent an increasing proportion of the published literature. This allows researchers to conduct focused meta-analytic reviews restricting the sample to studies with key design features (e.g., active control conditions that are intended to be therapeutic) [7]. Indeed, in the current sample which included 164 unique comparisons, 67 comparison (40.85%) were with an active therapy. Therefore, firm conclusions can still be made based on a restricted portion of studies. Further, meta-analytic methods allow studies to be weighted by sample size, in part ameliorating the draw back of underpowered individual studies (although, as discussed below, not ameliorating the drawback of publication bias) [15].

Indeed, the increased rate of publication in this area is perhaps part of what is driving the modest methodological progress, with smaller and more poorly designed studies conducted even in the absence of research funding. Increased publication pressure may further contribute, with scientists incentivized to publish lower quality research in pursuit of higher productivity [33]. Budgetary constraints and the pressure to publish cannot account for the lack of improvement across all six areas assessed, however. While some features likely do require larger financial resources (e.g., including active control conditions, larger samples, follow-up assessments), other features are more closely linked with design choices and reporting practices (e.g., treatment fidelity assessment, reporting of instructor training, reporting of ITT analyses). Along these lines, it was encouraging to see an increase of ITT analyses at least in the RCTS conducted in Europe and North America.

The phase of research being conducted may also have impacted the findings. As Dimidjian and Segal (2015) [34] call attention to, a large number of mindfulness-based interventions have been developed in recent years (through Stage I intervention generation / refinement studies). It is reasonable that these treatments would be tested initially in less rigorous designs (e.g., Stage II efficacy in research clinic trials using waitlist or treatment-as-usual comparisons). It is possible that in the coming years a larger number of more rigorous designs will appear as the creation of novel mindfulness-based treatment approaches is supplanted by more rigorous testing of established mindfulness therapies.

It is important to acknowledge that a relative lack of increased methodological quality over time may also not be unique to mindfulness research. Criticisms of the low statistical power in psychological research voiced by Cohen (1962) [35] rang true when reassessed 20 years later [36]. Concerns regarding sample size and reproducibility are highly visible across psychology and medicine in recent years as well [37–39].

Interestingly, results looked more encouraging when Teasdale et al. (2000) [28] was excluded. It appears that this study, which included several recommended design features (e.g., fidelity assessment, reporting of ITT analyses, large sample size), exerted a strong influence within the regression models, with significant improvements seen in several areas when the study was excluded. Nonetheless, several design features still did not show improvement (i.e., strength of the comparison type, length of follow-up, reporting of instructor training).

Given formal assessment of the influence of publication bias (e.g., funnel plots) was not feasible in the current design (as meta-analytic methods were not used), it is worth considering how the selective reporting of findings may have influenced our results. For some design features, such as sample size, it is likely that the exclusion of unpublished studies exerted a conservative influence. As sample size is directly linked with statistical power, smaller studies are less likely to detect significant effects, and therefore less likely to be published. If this research had been published, their inclusion would have made it less likely that increases in sample size would have been detected. Other design features are likely unrelated to the likelihood that a study is published (e.g., reporting of instructor training, assessment of fidelity). Conversely some design features may make it more likely that results are null (e.g., use of active control conditions), and thus less competitive for publication. Clearly the issue of publication bias is a significant one facing psychology and the sciences generally [38,39]. Here we will simply add our voice to the chorus calling for greater transparency in clinical trials reporting (e.g., through registering at clinicaltrials.gov) [15] and the publication of results disconfirming the authors’ hypotheses [40].

While we feel the current systematic review most directly examines changes over time in methodological quality of mindfulness research, our study is not without its shortcomings. First, the number of available studies included may have limited our ability to detect changes over time. It is worth noting that the direction of change of several of the design features assessed shows a shift towards improved quality; it is possible that a systematic review with a larger number of individual trials (and greater statistical power) would detect significant improvement in areas that we did not. A second limitation was weighting large and small studies equally. Unlike a traditional meta-analysis, our analyses were not weighted by sample size. As our interest was in the quality of study design, it seemed important to allow all studies to contribute equally (and not to bias results through giving more weight to larger and potentially better designed studies). A third limitation was not analyzing rates of clinicaltrials.gov registration as an additional desirable study design feature (a feature which greatly enhances the transparency of clinical trials research through requiring preregistration of study hypotheses, planned analyses, and outcomes). We also did not assess other potentially relevant design features, of which there are many (e.g., having study personnel, including data analysts, blinded to treatment condition; defining primary outcomes *a priori*) [26]. (An interesting future study could examine whether the six design features or other study quality indicators mentioned here predict treatment outcome.) We considered assessing rates of trial preregistration but chose not to do so both because this has not been a recommendation consistently voiced in the mindfulness literature and because, in contrast to medical journals, this has historically not been a requirement for most journals in psychology. A fourth limitation was restricting our sample to RCTs. This decision significantly limited the sample of studies that could have been included, and therefore may have both limited our statistical power to detect effects as well as limited our ability to detect changes in additional design features (e.g., the use of RCTs versus pre-post designs). A fifth related limitation was restricting our sample to clinical conditions, which likewise reduced the available number of studies. It may be that this choice exerted a conservative rather than a liberal bias on our ability to detect effects, with studies including clinical samples perhaps more likely to include more rigorous design features. It would be worthwhile examining whether the trends reported here are replicated in studies conducted in non-clinical samples. Lastly, our review relied exclusively on information reported in the published manuscript. It is possible that some design features (e.g., fidelity assessment) could have occurred and were simply not reported.

In conclusion, the 16 years of mindfulness research reviewed here provided modest evidence that the quality of research is improving over time. There may be various explanations for this (e.g., an increasing number of novel mindfulness-based interventions being first tested in less rigorous designs; the undue influence of early, high-quality studies). However, it is our hope that demonstrating this fact empirically will encourage future researchers to work towards the recommendations here and ultimately towards a clearer and scientifically-informed understanding of the potential and limitations of these treatments.

## Supporting information

S1 Table List of disorders and recognized evidence-based treatments. (DOCX)

S2 Table Characteristics of included studies. (DOCX)

S3 Table Included studies. (DOCX)

S4 Table PRISMA checklist. (DOC)
