# Supplementary material for: Is mindfulness research methodology improving over time? A systematic review
Source: PLoS One. 2017 Oct 31;12(10):e0187298. doi: 10.1371/journal.pone.0187298 (PMC5663486; doi:10.1371/journal.pone.0187298)
Supplement: S1 Table — (DOCX) [file pone.0187298.s001.docx]

S1 Table. List of disorders and recognized evidence-based treatments.

| APA Large Category | Collapsed Disorder Category | Subcategory | Treatments |
| --- | --- | --- | --- |
| ADHD | ADHD |  | CBT |
| Bipolar | Bipolar |  | Psychoed, Systematic Care, Cog Ther, Family-Focused Ther, Interpersonal and Social Rhythm Ther |
| Borderline PD | Borderline |  | DBT, Mentalization-Based Therapy, Schema-Focused Ther, Transference-Focused Ther |
| Child and Adolescent Disorders |  |  | NA |
| Chronic or Persistent Pain | Pain | Fibromyalgia | Multicomponent CBT for FM |
| Chronic or Persistent Pain | Pain | Chronic low back pain | Behavioral Therapy, CBT for CLBP |
| Chronic or Persistent Pain | Pain | Rheumatologic pain | Multicomponent CBT for Rheumatic Pain |
| Chronic or Persistent Pain | Pain | Headache | CBT for Chronic Headache |
| Chronic or Persistent Pain | Pain | Chronic or persistent pain | ACT for Chronic Pain |
| Depression | Depression |  | BT/BA, CT, Cog Behav Analysis System of Psychotherapy, IPT, Problem-Solving Ther, Self-Management / Self-Control Therapy, ACT, Behavioral Couple Ther, EFT, REBT, Reminiscence / Life Review Therapy, Self-System Therapy, Short-Term Psychodynamic Therapy |
| Eating Disorders and Obesity | Weight/Eating | Anorexia Nervosa | Family-Based Treatment, CBT |
| Eating Disorders and Obesity | Weight/Eating | Bulimia Nervosa | CBT, IPT, Family-Based Treatment, Healthy-Weight Program |
| Eating Disorders and Obesity | Weight/Eating | Binge Eating Disorder | CBT, IPT |
| Eating Disorders and Obesity | Weight/Eating | Obesity and Pediatric Overweight | Behavioral Weight Loss Treatment |
| Generalized Anxiety Disorder | Anxiety |  | CBT |
| Insomnia | Sleep |  | CBT, Sleep Restriction Therapy, Stimulus Control Therapy, Relaxation Training, Paradoxical Intention, EMG Biofeedback |
| Mixed Anxiety | Anxiety |  | ACT for Mixed Anxiety Conditions |
| OCD | Anxiety |  | Exposure and Response Prevention, CT, ACT |
| Panic Disorder | Anxiety |  | CBT, Applied Relaxation, Psychoanalytic Treatment |
| PTSD | PTSD |  | PE, PCT, CPT, Seeking Safety, Stress Inoculation Therapy, EMDR, Psych Debriefing |
| Schizophrenia and Other Mental Illnesses | Schizophrenia |  | Social Skills Training, CBT, Assertive Community Treatment, Family Psychoed, Supported Employment, Social learning / Token Economy Programs, Cog Remediation, ACT for Psychosis, Cog Adaptation Training, Illness Management and Recovery |
| Social Phobia and Public Speaking Anxiety | Anxiety |  | CBT |
| Specific Phobias | Anxiety |  | Exposure therapies |
| Substance and Alcohol Use Disorders | Addiction | Mixed substance abuse | MI, MET, MET + CBT, Prize-Based Contingency Management, Seeking Safety, Friends Care, Guided Self-Change |
| Substance and Alcohol Use Disorders | Addiction | Alcohol | Behavioral Couples Therapy for Alcohol Use Disorders, Moderate Drinking, Prize-Based Contingency Management |
| Substance and Alcohol Use Disorders | Addiction | Cocaine | Prize-Based Contingency Management |
| Substance and Alcohol Use Disorders | Smoking | Smoking | Smoking Cessation with Weight Gain Prevention |
|  |  |  |  |
| Other society's treatments |  |  |  |
|  |  | Smoking | Freedom From Smoking (American Lung Association), CBT (U.S. Department of Health and Human Services) |
|  |  | Addiction | CBT (National Institute of Drug Abuse) |

Note: APA = American Psychological Association; ADHD = Attention Deficit Hyperactivity Disorder; CBT = Cognitive Behavioral Therapy; Psychoed = Psychoeducation; PD = Personality Disorder; Cog = Cognitive; Ther = Therapy; FM = Fibromyalgia; CLBP = Chronic Low Back Pain; BT = Behavioral Therapy; BA = Behavioral Activation; CT = Cognitive Therapy; EFT = Emotion-Focused Therapy; REBT = Rational Emotive Behavior Therapy; IPT = Interpersonal Psychotherapy; EMG = Electromyography; OCD = Obsessive-Compulsive Disorder; ACT = Acceptance and Commitment Therapy; PTSD = Posttraumatic Stress Disorder; PE = Prolonged Exposure; PCT = Present-Centered Therapy; CPT = Cognitive Processing Therapy; EMDR = Eye Movement Desensitization and Reprocessing; MI = Motivational Interviewing; MET = Motivational Enhancement Therapy; U.S. = United States.
