# Supplementary material for: Is mindfulness research methodology improving over time? A systematic review
Source: PLoS One. 2017 Oct 31;12(10):e0187298. doi: 10.1371/journal.pone.0187298 (PMC5663486; doi:10.1371/journal.pone.0187298)
Supplement: S2 Table — (DOCX) [file pone.0187298.s002.docx]

S2 Table. Characteristics of included studies.

| Original Study | Diagnosis | Mindful | Cont | Age | Percent Female | | Percent College | | Country | Tx n | | | Cont n | FU | | Fidel | | Train | | ITT | | Obj | | Time Match | |
| --- | --- | --- | --- | --- | --- | --- | --- | --- | --- | --- | --- | --- | --- | --- | --- | --- | --- | --- | --- | --- | --- | --- | --- | --- | --- |
| Abolghasemi 2015 | Depression | MBCT | EBT | 29 | 60 | NA | | Iran | | | 15 | 15 | | 2 | No | | No | | Yes | | No | | Yes | |  |
| Alberts 2010 | Weight/Eating | None | No tx | 51.88 | 89.47 | NA | | Netherlands | | | 10 | 9 | | NA | No | | No | | Yes | | Yes | | No | |  |
| Alberts 2012 | Weight/Eating | MBCT | No tx | 48.5 | 100 | NA | | Netherlands | | | 12 | 14 | | NA | No | | No | | Yes | | Yes | | No | |  |
| Alexander 2012 | Depression | None | EBT | 45 | 72 | 66 | | United States | | | 67 | 67 | | 2 | No | | No | | No | | No | | Yes | |  |
| Alterman 2004 | Addiction | None | No tx | 36.48 | 54.85 | NA | | United States | | | 18 | 13 | | 3 | No | | No | | No | | Yes | | No | |  |
| Arch 2013 | Anxiety | MBSR | EBT | 45.91 | 17 | NA | | United States | | | 45 | 60 | | 3 | Yes | | Prot spec | | Yes | | Yes | | Yes | |  |
| Asl 2014 | Depression | MBCT | No tx | 29.5 | 0 | NA | | Iran | | | 18 | 17 | | NA | No | | No | | No | | No | | No | |  |
| Astin 2003 | Pain | MBSR | Spec | 47.7 | 99.2 | 17.2 | | United States | | | 64 | 64 | | 3.72 | No | | No | | No | | Yes | | Yes | |  |
| Atkinson 2016 | Weight/Eating | MBCT | Spec | 20.57 | 100 | NA | | Australia | | | 17 | 16 | | 6 | Yes | | No | | No | | No | | Yes | |  |
| Atkinson 2016 | Weight/Eating | MBCT | No tx | 20.57 | 100 | NA | | Australia | | | 17 | 17 | | 6 | Yes | | No | | No | | No | | No | |  |
| Bakhshani 2016 | Pain | MBSR | No tx | 31.05 | 67.5 | NA | | Iran | | | 20 | 20 | | NA | No | | No | | No | | No | | No | |  |
| Banth 2015 | Pain | MBSR | No tx | 37.5 | 100 | NA | | Iran | | | 39 | 48 | | 1 | No | | No | | No | | No | | No | |  |
| Barnhofer 2009 | Depression | MBCT | No tx | 41.93 | 67.86 | NA | | UK | | | 16 | 15 | | NA | No | | Prot spec | | Yes | | Yes | | No | |  |
| Bedard 2014 | Depression | MBCT | No tx | 46.77 | 45 | 61 | | Canada | | | 57 | 48 | | NA | No | | No | | No | | No | | No | |  |
| Black 2015 | Sleep | None | Spec | 66.3 | 67 | NA | | United States | | | 24 | 25 | | NA | No | | Prot spec | | Yes | | No | | Yes | |  |
| Bondolfi 2010 | Depression | MBCT | No tx | 47.45 | 71.58 | NA | | Switzerland | | | 31 | 29 | | 12 | Yes | | Prot spec | | Yes | | Yes | | No | |  |
| Bowen 2009 | Addiction | MBRP | Spec | 40.5 | 36.3 | NA | | United States | | | 93 | 70 | | 4 | No | | Prot spec | | No | | No | | Yes | |  |
| Bowen 2014 | Addiction | MBRP | Spec | 38.41 | 29.74 | 45.46 | | United States | | | 103 | 95 | | 10 | Yes | | Prot spec | | No | | Yes | | Yes | |  |
| Bowen 2014 | Addiction | MBRP | Spec | 38.41 | 29.74 | 45.46 | | United States | | | 103 | 88 | | 10 | Yes | | Prot spec | | No | | Yes | | Yes | |  |
| Brewer 2009 | Addiction | MBRP | EBT | 38.2 | 28 | 52 | | United States | | | 21 | 15 | | NA | No | | Not prot spec | | No | | No | | Yes | |  |
| Brewer 2011 | Smoking | MBRP | EBT | 45.9 | 37.9 | 57.4 | | United States | | | 41 | 47 | | 3 | No | | Not prot spec | | Yes | | Yes | | Yes | |  |
| Brown 2013 | Pain | None | No tx | 46.61 | 75 | NA | | UK | | | 20 | 20 | | NA | No | | Prot spec | | No | | No | | No | |  |
| Cash 2015 | Pain | MBSR | No tx | NA | 100 | NA | | United States | | | 51 | 40 | | 2 | No | | Prot spec | | Yes | | No | | No | |  |
| Cathcart 2014 | Pain | MBSR/MBCT | No tx | 45.54 | 62.75 | NA | | Australia | | | 29 | 29 | | NA | No | | Prot spec | | No | | Yes | | No | |  |
| Chacko 2016 | Weight/Eating | MBSR | Min tx | 53.95 | 84 | 83.5 | | United States | | | 9 | 9 | | 3.21 | No | | Prot spec | | Yes | | Yes | | No | |  |
| Chadwick 2009 | Schizophrenia | None | No tx | 41.6 | NA | NA | | UK | | | 11 | 11 | | NA | No | | Prot spec | | No | | No | | No | |  |
| Chadwick 2016 | Schizophrenia | None | No tx | 42 | 50 | 28.5 | | UK | | | 54 | 54 | | 6 | Yes | | Prot spec | | Yes | | Yes | | No | |  |
| Chavooshi 2016 | Pain | MBSR | Spec | 32.67 | 30 | NA | | Iran | | | 20 | 23 | | 3 | No | | No | | No | | No | | Yes | |  |
| Chavooshi 2016 | Pain | MBSR | No tx | 32.67 | 30 | NA | | Iran | | | 20 | 20 | | 3 | No | | No | | No | | No | | No | |  |
| Cherkin 2016 | Pain | MBSR | No tx | 49.3 | 65.7 | 92.3 | | United States | | | 116 | 113 | | 10 | Yes | | Prot spec | | Yes | | No | | No | |  |
| Cherkin 2016 | Pain | MBSR | EBT | 49.3 | 65.7 | 92.3 | | United States | | | 116 | 113 | | 10 | Yes | | Prot spec | | Yes | | No | | Yes | |  |
| Chien 2013 | Schizophrenia | MBSR | No tx | 25.8 | 45 | NA | | China | | | 48 | 48 | | 15 | No | | No | | Yes | | Yes | | No | |  |
| Chien 2014 | Schizophrenia | MBSR | No tx | 25.63 | 42.99 | 23.37 | | China | | | 36 | 35 | | 24 | Yes | | Prot spec | | Yes | | Yes | | No | |  |
| Chien 2014 | Schizophrenia | MBSR | Spec | 25.63 | 42.99 | 23.37 | | China | | | 36 | 36 | | 24 | Yes | | Prot spec | | Yes | | Yes | | Yes | |  |
| Chiesa 2012 | Depression | MBCT | Spec | NA | 75 | 0.25 | | Italy | | | 9 | 9 | | NA | No | | Prot spec | | No | | Yes | | Yes | |  |
| Chiesa 2015 | Depression | MBCT | Spec | 48.95 | 72.09 | 35 | | Italy | | | 26 | 24 | | 4.19 | No | | Prot spec | | Yes | | Yes | | Yes | |  |
| Colgan 2016 | PTSD | MBSR | Spec | 52 | 5.88 | NA | | United States | | | 28 | 28 | | NA | Yes | | No | | No | | No | | Yes | |  |
| Colgan 2016 | PTSD | MBSR | Non-spec | 52 | 5.88 | NA | | United States | | | 28 | 28 | | NA | Yes | | No | | No | | No | | Yes | |  |
| Corsica 2014 | Weight/Eating | MBSR | Spec | 45.4 | 98 | NA | | United States | | | 19 | 20 | | 1.5 | No | | Prot spec | | No | | Yes | | Yes | |  |
| Crane 2008 | Depression | MBCT | No tx | 44.96 | NA | 66.67 | | UK | | | 33 | 35 | | NA | No | | Prot spec | | No | | No | | No | |  |
| Daubenmier 2011 | Weight/Eating | MBSR/MBCT/MB-EAT | No tx | 40.89 | 100 | NA | | United States | | | 24 | 23 | | NA | No | | No | | Yes | | Yes | | No | |  |
| Daubenmier 2016 | Weight/Eating | MBSR/MB-EAT | Spec | 47.49 | 82.39 | 64.81 | | United States | | | 100 | 94 | | 12.5 | No | | Not prot spec | | Yes | | Yes | | Yes | |  |
| Davis 2013 | Smoking | MTS | EBT | 21.93 | 29.1 | NA | | United States | | | 30 | 25 | | NA | No | | No | | Yes | | Yes | | Yes | |  |
| Davis-JSAT 2014 | Smoking | MTS | EBT | 44.69 | 48 | 59.9 | | United States | | | 68 | 67 | | 5 | No | | Prot spec | | Yes | | Yes | | Yes | |  |
| Davis-SUM 2014 | Smoking | MTS | Min tx | 41.65 | 50 | 45.41 | | United States | | | 105 | 91 | | 5 | No | | Prot spec | | Yes | | Yes | | No | |  |
| Day 2014 | Pain | MBCT | No tx | 41.7 | 88.9 | NA | | United States | | | 19 | 17 | | NA | Yes | | Prot spec | | Yes | | No | | No | |  |
| DeDios 2012 | Addiction | None | No tx | 23.03 | 100 | NA | | United States | | | 22 | 12 | | 2 | Yes | | Prot spec | | No | | No | | No | |  |
| DeJong 2016 | Depression | MBCT | No tx | 50.7 | 72.7 | NA | | United States | | | 26 | 14 | | NA | No | | No | | No | | Yes | | No | |  |
| DeJong 2016 | Pain | MBCT | No tx | 50.7 | 72.7 | NA | | United States | | | 26 | 14 | | NA | No | | No | | No | | Yes | | No | |  |
| Delgado 2010 | Anxiety | None | Spec | 21 | 100 | 100 | | Spain | | | 18 | 18 | | NA | No | | No | | No | | No | | Yes | |  |
| Dimidjian 2016 | Depression | MBCT | Min tx | 29.85 | 100 | 76.75 | | United States | | | 43 | 43 | | 12.9 | Yes | | Prot spec | | Yes | | Yes | | No | |  |
| Eisendrath 2016 | Depression | MBCT | Spec | 46.16 | 76.3 | NA | | United States | | | 87 | 86 | | 10 | Yes | | Prot spec | | Yes | | Yes | | Yes | |  |
| Esmer 2010 | Pain | MBSR | No tx | 55.08 | 44 | NA | | United States | | | 19 | 21 | | NA | No | | Prot spec | | No | | No | | No | |  |
| Fissler 2016 | Depression | MBCT | Spec | 41.55 | 60.29 | NA | | Germany | | | 38 | 36 | | NA | No | | No | | No | | No | | Yes | |  |
| Fleer 2014 | Depression | MBCT | No tx | 40.5 | 73.95 | 65.15 | | Netherlands | | | 23 | 23 | | 5.76 | No | | No | | Yes | | No | | No | |  |
| Fogarty 2015 | Pain | MBSR | No tx | 53.5 | 88.1 | NA | | New Zealand | | | 26 | 25 | | 4 | No | | No | | No | | No | | No | |  |
| Garland 2010 | Addiction | MORE | Spec | 40.3 | 20.8 | NA | | United States | | | 27 | 26 | | NA | No | | Not prot spec | | No | | No | | Yes | |  |
| Garland-JCCP 2014 | Pain | MORE | Spec | 48.34 | 67.83 | 70.43 | | United States | | | 57 | 58 | | 3 | Yes | | Prot spec | | No | | No | | Yes | |  |
| Garland-JCO 2014 | Sleep | MBSR | EBT | 58.89 | 72 | NA | | Canada | | | 64 | 47 | | 3 | No | | Prot spec | | Yes | | Yes | | Yes | |  |
| Garland 2016 | Addiction | MORE | EBT | 37.56 | 100 | NA | | United States | | | 64 | 64 | | NA | Yes | | Prot spec | | Yes | | No | | Yes | |  |
| Garland 2016 | Addiction | MORE | Spec | 37.56 | 100 | NA | | United States | | | 64 | 52 | | NA | Yes | | Prot spec | | Yes | | No | | Yes | |  |
| Geschwind 2011 | Depression | MBCT | No tx | 43.88 | 75.93 | NA | | Netherlands | | | 64 | 66 | | NA | No | | Prot spec | | Yes | | Yes | | No | |  |
| Glasner 2016 | Addiction | MBRP | Non-spec | 45.3 | 28.6 | NA | | United States | | | 31 | 32 | | 1 | Yes | | Prot spec | | No | | Yes | | Yes | |  |
| Godfrin 2010 | Depression | MBCT | No tx | 45.66 | 81.13 | 70 | | Belgium | | | 52 | 54 | | 12 | No | | Not prot spec | | Yes | | Yes | | No | |  |
| Goldin 2016 | Anxiety | MBSR | No tx | 32.7 | 55.6 | NA | | United States | | | 36 | 36 | | 12 | Yes | | Prot spec | | Yes | | No | | No | |  |
| Goldin 2016 | Anxiety | MBSR | EBT | 32.7 | 55.6 | NA | | United States | | | 36 | 36 | | 12 | Yes | | Prot spec | | Yes | | No | | Yes | |  |
| Greenberg 2016 | Depression | MBCT | No tx | 38.47 | 62.15 | NA | | United States | | | 22 | 18 | | NA | No | | Prot spec | | No | | Yes | | No | |  |
| Gross 2011 | Sleep | MBSR | EBT | 49.17 | 73.33 | 80 | | United States | | | 20 | 10 | | 3 | No | | Prot spec | | No | | Yes | | No | |  |
| Hanstede 2008 | Anxiety | None | No tx | 25.7 | 70.59 | 100 | | Netherlands | | | 8 | 9 | | NA | No | | No | | No | | No | | No | |  |
| Helmes 2015 | Anxiety | MBCT | Non-spec | 83 | NA | NA | | Australia | | | 26 | 26 | | 1 | Yes | | Prot spec | | Yes | | No | | Yes | |  |
| Hepark 2015 | ADHD | MBCT | No tx | 35.89 | 54.54 | NA | | Netherlands | | | 55 | 48 | | NA | No | | Prot spec | | No | | Yes | | No | |  |
| Hoge 2013 | Anxiety | MBSR | Spec | 39.16 | 50.56 | NA | | United States | | | 48 | 45 | | NA | Yes | | Prot spec | | Yes | | Yes | | Yes | |  |
| Huijbers 2015 | Depression | MBCT | No tx | 51.75 | 71.97 | NA | | Netherlands | | | 33 | 35 | | 13 | Yes | | Not prot spec | | Yes | | Yes | | No | |  |
| Imani 2015 | Addiction | MBRP | No tx | 37.41 | 3.4 | 7.5 | | Iran | | | 15 | 15 | | NA | No | | No | | No | | Yes | | No | |  |
| Iranshahri 2015 | Addiction | MBSR | No tx | 29.46 | 0 | NA | | Iran | | | 15 | 15 | | NA | No | | No | | Yes | | No | | No | |  |
| Jay 2015 | Pain | None | No tx | 46.55 | 100 | NA | | Denmark | | | 56 | 56 | | NA | No | | Not prot spec | | Yes | | No | | No | |  |
| Jazaieri 2012 | Anxiety | MBSR | Spec | 32.87 | 51.79 | NA | | United States | | | 31 | 25 | | 3 | No | | Prot spec | | No | | No | | Yes | |  |
| Johannsen 2016 | Pain | MBCT | No tx | 56.75 | 100 | 0.6 | | Denmark | | | 67 | 62 | | 6 | No | | Prot spec | | No | | No | | No | |  |
| Kanter 2016 | Pain | MBSR | No tx | 45.26 | 100 | 90 | | United States | | | 9 | 11 | | NA | No | | Prot spec | | No | | No | | No | |  |
| Kaviani 2012 | Depression | MBCT | No tx | 21.7 | 100 | 100 | | Iran | | | 15 | 15 | | 6 | Yes | | Prot spec | | No | | No | | No | |  |
| Kearney 2013 | PTSD | MBSR | No tx | 52 | 21.28 | NA | | United States | | | 25 | 22 | | 4 | No | | Prot spec | | Yes | | No | | No | |  |
| Keune 2011 | Depression | MBCT | No tx | 47.16 | 74.03 | NA | | Germany | | | 45.5 | 45.5 | | NA | No | | Prot spec | | No | | No | | No | |  |
| King 2016 | PTSD | MBCT | EBT | 32.13 | 0 | 78.26 | | United States | | | 26 | 17 | | NA | No | | Prot spec | | No | | Yes | | Yes | |  |
| Kocovski 2013 | Anxiety | MBCT | EBT | 34 | 54 | NA | | Canada | | | 53 | 53 | | NA | Yes | | Prot spec | | Yes | | Yes | | Yes | |  |
| Kocovski 2013 | Anxiety | MBCT | No tx | 34 | 54 | NA | | Canada | | | 53 | 31 | | NA | Yes | | Prot spec | | Yes | | Yes | | No | |  |
| Koszycki 2007 | Anxiety | MBSR | EBT | 38.24 | 52.83 | NA | | Canada | | | 26 | 27 | | NA | Yes | | Prot spec | | Yes | | Yes | | Yes | |  |
| Koszycki 2016 | Anxiety | None | No tx | 39.77 | 79.54 | NA | | Canada | | | 21 | 18 | | NA | No | | Prot spec | | Yes | | Yes | | No | |  |
| Kristeller 2013 | Weight/Eating | MB-EAT | No tx | 46.55 | 88 | NA | | United States | | | 53 | 47 | | 4 | Yes | | No | | No | | Yes | | No | |  |
| Kristeller 2013 | Weight/Eating | MB-EAT | Spec | 46.55 | 88 | NA | | United States | | | 53 | 50 | | 4 | Yes | | No | | No | | Yes | | Yes | |  |
| Kuyken 2008 | Depression | MBCT | EBT | 49.16 | 76.5 | 21.51 | | UK | | | 61 | 62 | | 13 | Yes | | Prot spec | | Yes | | Yes | | No | |  |
| Kuyken 2015 | Depression | MBCT | EBT | 49.5 | 76.5 | 74 | | UK | | | 212 | 212 | | 23 | Yes | | Prot spec | | Yes | | Yes | | No | |  |
| LaCour 2015 | Pain | MBSR | No tx | 47.69 | 85.32 | 27.52 | | Denmark | | | 54 | 55 | | NA | No | | Prot spec | | Yes | | No | | No | |  |
| Langer 2010 | Schizophrenia | MBCT | Non-spec | 21.29 | 84.21 | 100 | | Spain | | | 18 | 20 | | NA | No | | No | | No | | No | | Yes | |  |
| Langer 2012 | Schizophrenia | MBCT | No tx | 34.21 | 41.26 | NA | | Spain | | | 11 | 12 | | NA | No | | Not prot spec | | No | | No | | No | |  |
| Lee 2011 | Addiction | MBRP | No tx | 40.7 | 0 | NA | | Taiwan | | | 10 | 14 | | NA | No | | Prot spec | | Yes | | No | | No | |  |
| Lopez-Navarro 2015 | Schizophrenia | None | No tx | 38.84 | 17.2 | NA | | Spain | | | 22 | 22 | | NA | No | | Not prot spec | | Yes | | Yes | | No | |  |
| Ma 2004 | Depression | MBCT | No tx | 44.52 | 76.04 | NA | | UK | | | 37 | 38 | | 12 | No | | Prot spec | | Yes | | Yes | | No | |  |
| Madani 2013 | Anxiety | None | No tx | NA | 100 | 54.16 | | Iran | | | 15 | 15 | | 2 | No | | No | | No | | No | | No | |  |
| Majid 2012 | Anxiety | MBSR | No tx | 32.19 | NA | NA | | Iran | | | 17 | 16 | | NA | No | | Prot spec | | Yes | | No | | No | |  |
| Manicavasgar 2011 | Depression | MBCT | EBT | 45.84 | 64.44 | NA | | Australia | | | 30 | 39 | | 12 | Yes | | Not prot spec | | No | | No | | Yes | |  |
| Mann 2016 | Depression | MBCT | No tx | 36.2 | 95 | 92.5 | | UK | | | 19 | 19 | | 7 | Yes | | Prot spec | | No | | No | | No | |  |
| McIndoo 2016 | Depression | MBSR | EBT | 19.22 | 62.04 | 100 | | United States | | | 20 | 16 | | 1 | Yes | | Prot spec | | Yes | | Yes | | Yes | |  |
| McIndoo 2016 | Depression | MBSR | No tx | 19.22 | 62.04 | 100 | | United States | | | 20 | 14 | | 1 | Yes | | Prot spec | | Yes | | Yes | | No | |  |
| Meadows 2014 | Depression | MBCT | No tx | 48.35 | 81.28 | 55.64 | | Australia | | | 102 | 102 | | 24 | Yes | | Prot spec | | Yes | | Yes | | No | |  |
| Michalak 2015 | Depression | MBCT | No tx | 50.84 | 62.26 | NA | | Germany | | | 36 | 35 | | 6.33 | Yes | | Prot spec | | Yes | | Yes | | No | |  |
| Michalak 2015 | Depression | MBCT | EBT | 50.84 | 62.26 | NA | | Germany | | | 36 | 35 | | 6.33 | Yes | | Prot spec | | Yes | | Yes | | Yes | |  |
| Miller 2014 | Weight/Eating | MB-EAT | Spec | 53.95 | 63.48 | 53.87 | | United States | | | 32 | 36 | | 3 | Yes | | Not prot spec | | No | | Yes | | Yes | |  |
| Mitchell 2013 | ADHD | None | No tx | 38.6 | 60 | 85 | | United States | | | 11 | 11 | | NA | No | | No | | No | | Yes | | No | |  |
| Moore 2016 | Anxiety | MBSR | Spec | 70.95 | 75.57 | NA | | United States | | | 32 | 35 | | NA | No | | No | | No | | No | | Yes | |  |
| Morone 2008 | Pain | MBSR | No tx | 74.9 | 57 | 59.46 | | United States | | | 19 | 18 | | NA | No | | Prot spec | | Yes | | No | | No | |  |
| Morone 2009 | Pain | MBSR | Spec | 75.29 | 62.86 | 77.14 | | United States | | | 20 | 20 | | 4 | No | | Prot spec | | No | | No | | Yes | |  |
| Morone 2016 | Pain | MBSR | Spec | 74.5 | 66.3 | 75.5 | | United States | | | 140 | 142 | | 6 | No | | No | | Yes | | No | | Yes | |  |
| Nakamura 2013 | Sleep | MBSR | Spec | 51.18 | 78.95 | NA | | United States | | | 20 | 18 | | 2 | No | | Prot spec | | Yes | | No | | Yes | |  |
| Nassif 2016 | Pain | None | No tx | 47.45 | 0 | NA | | United States | | | 6.5 | 6.5 | | NA | No | | No | | No | | No | | No | |  |
| Niles 2012 | PTSD | None | Non-spec | 55.25 | 0 | NA | | United States | | | 17 | 16 | | 1.5 | Yes | | Prot spec | | No | | Yes | | Yes | |  |
| Omidi 2013 | Depression | MBCT | No tx | 28 | 66.33 | NA | | Iran | | | 30 | 30 | | NA | No | | No | | Yes | | No | | No | |  |
| Omidi 2013 | Depression | MBCT | EBT | 28 | 66.33 | NA | | Iran | | | 30 | 30 | | NA | No | | No | | Yes | | No | | Yes | |  |
| Ong 2014 | Sleep | MBSR | No tx | 43.77 | 71.46 | NA | | United States | | | 19 | 16 | | NA | Yes | | Prot spec | | Yes | | Yes | | No | |  |
| Panahi 2016 | Depression | MBCT | No tx | NA | 100 | 100 | | Iran | | | 30 | 30 | | NA | No | | Prot spec | | Yes | | No | | No | |  |
| Parra-Delgado 2013 | Pain | MBCT | No tx | 52.9 | 100 | NA | | Spain | | | 17 | 16 | | 3 | No | | Prot spec | | No | | No | | No | |  |
| Perich 2013 | Bipolar | MBCT | No tx | NA | 65.49 | 77.47 | | Australia | | | 48 | 47 | | 12 | Yes | | Prot spec | | Yes | | Yes | | No | |  |
| Piet 2010 | Anxiety | MBCT | EBT | 21.83 | 69.31 | NA | | Denmark | | | 14 | 12 | | NA | No | | Prot spec | | Yes | | Yes | | Yes | |  |
| Plews-Ogan 2005 | Pain | MBSR | Spec | 46.5 | NA | NA | | United States | | | 10 | 10 | | 1 | No | | No | | No | | No | | No | |  |
| Plews-Ogan 2005 | Pain | MBSR | No tx | 46.5 | NA | NA | | United States | | | 10 | 10 | | 1 | No | | No | | No | | No | | No | |  |
| Polusny 2015 | PTSD | MBSR | EBT | 58.5 | 16 | NA | | United States | | | 58 | 58 | | 2 | Yes | | Prot spec | | Yes | | Yes | | No | |  |
| Possemato 2016 | PTSD | MBSR | No tx | 46.4 | 12.9 | NA | | United States | | | 36 | 26 | | 1 | Yes | | Prot spec | | Yes | | Yes | | No | |  |
| Pots 2014 | Depression | MBCT | No tx | 47.93 | 78.1 | NA | | Netherlands | | | 76 | 75 | | NA | No | | Prot spec | | Yes | | No | | No | |  |
| Pradhan 2007 | Pain | MBSR | No tx | 54.48 | 87.3 | 61.9 | | United States | | | 31 | 32 | | 4 | No | | Prot spec | | No | | Yes | | No | |  |
| Rungreangkulkij 2011 | Depression | None | No tx | 48.5 | 93.75 | NA | | Thailand | | | 32 | 32 | | NA | No | | Prot spec | | Yes | | No | | No | |  |
| Schmidt 2011 | Pain | MBSR | No tx | 52.5 | 100 | 31.59 | | Germany | | | 59 | 59 | | 2 | No | | Prot spec | | Yes | | Yes | | No | |  |
| Schmidt 2011 | Pain | MBSR | Spec | 52.5 | 100 | 31.59 | | Germany | | | 59 | 59 | | 2 | No | | Prot spec | | Yes | | Yes | | Yes | |  |
| Schuver 2016 | Depression | MBSR | Spec | 42.68 | 100 | 92.5 | | United States | | | 20 | 20 | | 1 | No | | No | | No | | No | | Yes | |  |
| Segal 2010 | Depression | MBCT | EBT | 44 | 53 | NA | | Canada | | | 26 | 28 | | 16 | Yes | | Prot spec | | Yes | | Yes | | No | |  |
| Segal 2010 | Depression | MBCT | Non-spec | 44 | 53 | NA | | Canada | | | 26 | 30 | | 16 | Yes | | Prot spec | | Yes | | Yes | | No | |  |
| Shahar 2010 | Depression | MBCT | No tx | 46.6 | 84.44 | NA | | United States | | | 29 | 23 | | NA | No | | Prot spec | | No | | Yes | | No | |  |
| Shahar 2010 | Sleep | MBCT | No tx | 46.6 | 84.44 | NA | | United States | | | 29 | 23 | | NA | No | | Prot spec | | No | | Yes | | No | |  |
| Shallcross 2015 | Depression | MBCT | Spec | 34.85 | 77.17 | 95.65 | | United States | | | 46 | 46 | | 12 | Yes | | Prot spec | | Yes | | Yes | | Yes | |  |
| Singh 2014 | Smoking | None | No tx | 33.5 | 19.61 | NA | | Unknown | | | 25 | 26 | | 11 | Yes | | Not prot spec | | Yes | | No | | No | |  |
| Strauss 2012 | Depression | PBCT | No tx | 43 | 71.43 | NA | | UK | | | 14 | 14 | | NA | No | | Prot spec | | Yes | | No | | No | |  |
| Tang 2013 | Smoking | None | Spec | 21.46 | 29.63 | 100 | | United States | | | 15 | 12 | | NA | No | | No | | No | | Yes | | Yes | |  |
| Teasdale 2000 | Depression | MBCT | No tx | 43.32 | 75.9 | NA | | UK / Canada | | | 76 | 69 | | 12 | Yes | | Prot spec | | Yes | | Yes | | No | |  |
| Thompson 2010 | Depression | MBCT | No tx | 35.9 | 81 | 70 | | United States | | | 13 | 27 | | NA | No | | No | | No | | No | | No | |  |
| Tovote 2014 | Depression | MBCT | No tx | 53.1 | 49 | 25 | | Netherlands | | | 31 | 31 | | NA | Yes | | Prot spec | | Yes | | Yes | | No | |  |
| Tovote 2014 | Depression | MBCT | EBT | 53.1 | 49 | 25 | | Netherlands | | | 31 | 32 | | NA | Yes | | Prot spec | | Yes | | Yes | | Yes | |  |
| VanAalderen 2012 | Depression | MBCT | No tx | 47.5 | 71 | 59.47 | | Netherlands | | | 111 | 108 | | NA | No | | Prot spec | | No | | Yes | | No | |  |
| Vidrine 2016 | Smoking | MBCT | EBT | 48.7 | 54.9 | 66.3 | | United States | | | 154 | 155 | | 6 | No | | Prot spec | | Yes | | Yes | | Yes | |  |
| Vidrine 2016 | Smoking | MBCT | Min tx | 48.7 | 54.9 | 66.3 | | United States | | | 154 | 103 | | 6 | No | | Prot spec | | Yes | | Yes | | No | |  |
| Vollestad 2011 | Anxiety | MBSR | No tx | 42.5 | 67.11 | NA | | Norway | | | 39 | 37 | | NA | No | | No | | Yes | | No | | No | |  |
| Wang 2016 | Schizophrenia | MBSR | Spec | 24.4 | 47.83 | 19.6 | | China | | | 46 | 46 | | 6 | Yes | | Not prot spec | | No | | Yes | | Yes | |  |
| Wang 2016 | Schizophrenia | MBSR | No tx | 24.4 | 47.83 | 19.6 | | China | | | 46 | 46 | | 6 | Yes | | Not prot spec | | No | | Yes | | No | |  |
| Wells 2014 | Pain | MBSR | No tx | 45.57 | 89.47 | 94.74 | | United States | | | 10 | 9 | | 1 | No | | Prot spec | | Yes | | No | | No | |  |
| Williams 2008 | Depression | MBCT | No tx | 44.89 | 73.53 | 65 | | UK | | | 33 | 35 | | NA | No | | Prot spec | | No | | No | | No | |  |
| Williams 2014 | Depression | MBCT | No tx | 43 | 72 | NA | | UK | | | 108 | 56 | | 12 | Yes | | Prot spec | | No | | Yes | | No | |  |
| Williams 2014 | Depression | MBCT | Spec | 43 | 72 | NA | | UK | | | 108 | 110 | | 12 | Yes | | Prot spec | | No | | Yes | | Yes | |  |
| Witkiewitz 2014 | Addiction | MBRP | Spec | 34.2 | 100 | NA | | United States | | | 55 | 50 | | 3.49 | No | | Prot spec | | Yes | | No | | Yes | |  |
| Wong 2009 | Pain | MBSR | Spec | NA | NA | NA | | China | | | 50 | 50 | | 6 | No | | No | | No | | No | | Yes | |  |
| Wong 2011 | Pain | MBSR | Spec | 47.9 | NA | 24.24 | | China | | | 51 | 49 | | 6 | No | | Prot spec | | Yes | | No | | Yes | |  |
| Wong 2015 | Sleep | MBCT | Spec | 49.42 | 63.26 | NA | | Australia | | | 26 | 31 | | 3 | No | | Prot spec | | Yes | | Yes | | Yes | |  |
| Wong 2016 | Anxiety | MBCT | Spec | 50 | 79.1 | 33.7 | | China | | | 61 | 61 | | 9 | Yes | | Prot spec | | Yes | | No | | Yes | |  |
| Wong 2016 | Anxiety | MBCT | No tx | 50 | 79.1 | 33.7 | | China | | | 61 | 60 | | 9 | Yes | | Prot spec | | Yes | | No | | No | |  |
| Zangi 2012 | Pain | None | No tx | 53.9 | 78.9 | 52.1 | | Norway | | | 37 | 36 | | 8.53 | No | | Prot spec | | No | | No | | No | |  |
| Zautra 2008 | Depression | MBSR/MBCT | Non-spec | 54.26 | 67.57 | NA | | United States | | | 48 | 44 | | NA | Yes | | Prot spec | | Yes | | Yes | | Yes | |  |
| Zautra 2008 | Depression | MBSR/MBCT | EBT | 54.26 | 67.57 | NA | | United States | | | 48 | 52 | | NA | Yes | | Prot spec | | Yes | | Yes | | Yes | |  |
| Zautra 2008 | Pain | MBSR/MBCT | Non-spec | 54.26 | 67.57 | NA | | United States | | | 48 | 44 | | NA | Yes | | Prot spec | | Yes | | Yes | | Yes | |  |
| Zautra 2008 | Pain | MBSR/MBCT | EBT | 54.26 | 67.57 | NA | | United States | | | 48 | 52 | | NA | Yes | | Prot spec | | Yes | | Yes | | Yes | |  |
| Zemestani 2016 | Addiction | MBRP | Spec | 30.1 | 20.3 | 12.3 | | Iran | | | 37 | 37 | | 2 | Yes | | Prot spec | | Yes | | No | | Yes | |  |
| Zemestani 2016 | Depression | MBRP | Spec | 30.1 | 20.3 | 12.3 | | Iran | | | 37 | 37 | | 2 | Yes | | Prot spec | | Yes | | No | | Yes | |  |
| Zgierska 2016 | Pain | None | No tx | 51.8 | 80 | 77.1 | | United States | | | 21 | 14 | | 4.15 | No | | Not prot spec | | Yes | | No | | No | |  |
| Zhang 2015 | Sleep | MBSR | No tx | 78.1 | 41.67 | NA | | China | | | 30 | 30 | | NA | No | | Prot spec | | Yes | | No | | No | |  |

Note: Mindful = basis of mindfulness condition; Cont = strength of comparison condition; Percent college = percent of sample with some college; Tx n = intent-to-treat sample size for mindfulness condition (when reported); Cont n = intent-to-treat sample size for the control condition (when reported); FU = length of longest follow-up; Fidel = whether treatment fidelity was assessed; Train = whether instructor training in mindfulness was reported; ITT = whether intent-to-treat analysis was reported; Obj = whether an objective outcome measure was included; Time Match = whether treatment time was matched between the mindfulness and control condition; JSAT = Journal of Substance Abuse Treatment; SUM = Substance Use & Misuse; JCCP = Journal of Consulting and Clinical Psychology; JCO = Journal of Clinical Oncology; PTSD = Posttraumatic Stress Disorder; MBCT = Mindfulness-Based Cognitive Therapy; MBSR = Mindfulness-Based Stress Reduction; MBRP = Mindfulness-Based Relapse Prevention; MB-EAT = Mindfulness-Based Eating Awareness Training; MTS = Mindfulness Training for Smokers; MORE = Mindfulness-Oriented Recovery Enhancement; PBCT = Person-Based Cognitive Therapy; No tx = no treatment comparison condition; Min tx = minimal treatment comparison condition; Non-spec = non-specific active comparison condition; Spec = specific active comparison condition; EBT = Evidence-Based Treatment; NA = not applicable; prot spec = mindfulness protocol-specific training reported, not prot spec = training in mindfulness not specific to the mindfulness protocol reported. Studies are listed multiple times if they included multiple comparison groups and/or samples with comorbid conditions for which targeted outcomes were available.
