# Supplementary material for: Is mindfulness research methodology improving over time? A systematic review
Source: PLoS One. 2017 Oct 31;12(10):e0187298. doi: 10.1371/journal.pone.0187298 (PMC5663486; doi:10.1371/journal.pone.0187298)
Supplement: S3 Table — (DOCX) [file pone.0187298.s003.docx]

S3 Table. Included studies.

Abolghasemi A, Gholami H, Narimani M, Gamji M. The Effect of Beck’s Cognitive Therapy and Mindfulness-Based Cognitive Therapy on Sociotropic and Autonomous Personality Styles in Patients With Depression. *Iran J Psychiatry Behav Sci* 2015; **9:** e3665.

Alberts HJ, Mulkens S, Smeets M, Thewissen R. Coping with food cravings. Investigating the potential of a mindfulness-based intervention. *Appetite* 2010; **55:** 160–63.

Alberts HJ, Thewissen R, Raes L. Dealing with problematic eating behaviour. The effects of a mindfulness-based intervention on eating behaviour, food cravings, dichotomous thinking and body image concern. *Appetite* 2012; **58:** 847–51.

Alexander V, Tatum BC, Auth C, Takos D, Whittemore S, Fidaleo R. A study of mindfulness practices and cognitive therapy: Effects on depression and self-efficacy. *Int Journal Psychol Couns* 2012; **4:** 115–22.

Alterman AI, Koppenhaver JM, Mulholland E, Ladden LJ, Baime MJ. Pilot trial of effectiveness of mindfulness meditation for substance abuse patients. *J Subst Use* 2004; **9:** 259–68.

Arch JJ, Ayers CR, Baker A, Almklov E, Dean DJ, Craske MG. Randomized clinical trial of adapted mindfulness-based stress reduction versus group cognitive behavioral therapy for heterogeneous anxiety disorders. *Behav Res Ther* 2013; **51:** 185–96.

Asl NH, Barahmand U. Effectiveness of mindfulness-based cognitive therapy for co-morbid depression in drug-dependent males. *Arch Psychiatr Nurs* 2014; **28:** 314–8.

Astin JA, Berman BM, Bausell B, Lee WL, Hochberg M, Forys KL. The efficacy of mindfulness meditation plus Qigong movement therapy in the treatment of fibromyalgia: a randomized controlled trial. *J Rheumatol* 2003; **30:** 2257–62.

Atkinson MJ, Wade TD. Does mindfulness have potential in eating disorders prevention? A preliminary controlled trial with young adult women. *Early Interv Psychiatry* 2016; **10:** 234–245.

Bakhshani NM, Amirani A, Amirifard H, Shahrakipoor M. The effectiveness of mindfulness-based stress reduction on perceived pain intensity and quality of life in patients with chronic headache. *Glob J Health Sci* 2016; **8:** 142–51.

Banth S, Ardebil MD. Effectiveness of mindfulness meditation on pain and quality of life of patients with chronic low back pain. *Int J Yoga* 2015; **8:** 128–33.

Barnhofer T, Crane C, Hargus E, Amarasinghe M, Winder R, Williams JM. Mindfulness-based cognitive therapy as a treatment for chronic depression: a preliminary study. *Behav Res Ther* 2009; **47:** 366–73.

Barnhofer T, Crane C, Brennan K, et al. Mindfulness-based cognitive therapy (MBCT) reduces the association between depressive symptoms and suicidal cognitions in patients with a history of suicidal depression. *J Consult Clin Psychol* 2015; **83:** 1013–20.

Bédard M, Felteau M, Marshall S, et al. Mindfulness-based cognitive therapy reduces symptoms of depression in people with a traumatic brain injury: results from a randomized controlled trial. *J Head Trauma Rehab* 2014; **29:** E13–22.

Bieling PJ, Hawley LL, Bloch RT, et al. Treatment-specific changes in decentering following mindfulness-based cognitive therapy versus antidepressant medication or placebo for prevention of depressive relapse. *J Consult Clin Psychol* 2012; **80:** 365–72.

Black DS, O’Reilly GA, Olmstead R, Breen EC, Irwin MR. Mindfulness meditation and improvement in sleep quality and daytime impairment among older adults with sleep disturbances: a randomized clinical trial. *JAMA Intern Med* 2015; **175:** 494–501.

Bondolfi G, Jermann F, Van der Linden M, et al. Depression relapse prophylaxis with Mindfulness-Based Cognitive Therapy: replication and extension in the Swiss health care system. *J Affect Disorders* 2010; **122:** 224–31.

Bowen S, Chawla N, Collins SE, et al. Mindfulness-based relapse prevention for substance use disorders: a pilot efficacy trial. *Subst Abuse* 2009; **30:** 295–305.

Bowen S, Witkiewitz K, Clifasefi SL, et al. Relative efficacy of mindfulness-based relapse prevention, standard relapse prevention, and treatment as usual for substance use disorders: a randomized clinical trial. *JAMA Psychiatry* 2014; **71:** 547–56.

Brewer JA, Mallik S, Babuscio TA, et al. Mindfulness training for smoking cessation: results from a randomized controlled trial. *Drug Alcohol Depen* 2011; **119:** 72–80.

Brewer JA, Sinha R, Chen JA, et al. Mindfulness training and stress reactivity in substance abuse: results from a randomized, controlled stage I pilot study. *Subst Abuse* 2009; **30:** 306–17.

Britton WB, Haynes PL, Fridel KW, Bootzin RR. Polysomnographic and subjective profiles of sleep continuity before and after mindfulness-based cognitive therapy in partially remitted depression. *Psychosom Med* 2010; **72:** 539–48.

Britton WB, Haynes PL, Fridel KW, Bootzin RR. Mindfulness-based cognitive therapy improves polysomnographic and subjective sleep profiles in antidepressant users with sleep complaints. *Psychother Psychosom* 2012; **81:** 296–304.

Brown CA, Jones AK. Psychobiological correlates of improved mental health in patients with musculoskeletal pain after a mindfulness-based pain management program. *Clin J Pain* 2013; **29:** 233–44.

Cash E, Salmon P, Weissbecker I, et al. Mindfulness meditation alleviates fibromyalgia symptoms in women: results of a randomized clinical trial. *Ann Behav Med* 2015; **49:** 319–330.

Cathcart S, Barone V, Immink M, Proeve M. Mindfulness training does not reduce generalized hyperalgesia in chronic tension-type headache. *J Pain Manag* 2013; **6:** 217–221.

Cathcart S, Galatis N, Immink M, Proeve M, Petkov J. Brief mindfulness-based therapy for chronic tension-type headache: a randomized controlled pilot study. *Behav Cogn Psychother* 2014; **42:** 1–15.

Chacko SA, Yeh GY, Davis RB, Wee CC. A mindfulness-based intervention to control weight after bariatric surgery: Preliminary results from a randomized controlled pilot trial. *Complement Ther Med* 2016; **28:** 13–21.

Chadwick P, Hughes S, Russell D, Russell I, Dagnan D. Mindfulness groups for distressing voices and paranoia: a replication and randomized feasibility trial. *Behav Cogn Psychother* 2009; **37:** 403–12.

Chadwick P, Strauss C, Jones AM, et al. Group mindfulness-based intervention for distressing voices: a pragmatic randomised controlled trial. *Schizophr Res* 2016; **175:** 168–73.

Chavooshi B, Mohammadkhani P, Dolatshahee B. Efficacy of intensive short-term dynamic psychotherapy for medically unexplained pain: A pilot three-armed randomized controlled trial comparison with mindfulness-based stress reduction. *Psychother Psychosom* 2016; **85:** 123–5.

Cherkin DC, Sherman KJ, Balderson BH, et al. Effect of mindfulness-based stress reduction vs cognitive behavioral therapy or usual care on back pain and functional limitations in adults with chronic low back pain: A randomized clinical trial. *JAMA* 2016; **315:** 1240–9.

Chien WT, Lee IY. The mindfulness-based psychoeducation program for Chinese patients with schizophrenia. *Psych Serv* 2013; **64:** 376–9.

Chien WT, Thompson DR. Effects of a mindfulness-based psychoeducation programme for Chinese patients with schizophrenia: 2-year follow-up. *Br J Psychiatry* 2014; **205:** 52–59.

Chiesa A, Mandelli L, Serretti A. Mindfulness-based cognitive therapy versus psycho-education for patients with major depression who did not achieve remission following antidepressant treatment: a preliminary analysis. *J Altern Complement Med* 2012; **18:** 756–60.

Chiesa A, Castagner V, Andrisano C, et al. Mindfulness-based cognitive therapy vs. psycho-education for patients with major depression who did not achieve remission following antidepressant treatment. *Psychiatry Res* 2015; **226:** 474–83.

Colgan DD, Christopher M, Michael P, Wahbeh H. The Body Scan and Mindful Breathing Among Veterans with PTSD: Type of Intervention Moderates the Relationship Between Changes in Mindfulness and Post-treatment Depression. *Mindfulness* 2016; **7:** 372–83.

Collip D, Geschwind N, Peeters F, Myin-Germeys I, van Os J, Wichers M. Putting a hold on the downward spiral of paranoia in the social world: a randomized controlled trial of mindfulness-based cognitive therapy in individuals with a history of depression. *PLoS One* 2013; **8:** e66747.

Corsica J, Hood MM, Katterman S, Kleinman B, Ivan I. Development of a novel mindfulness and cognitive behavioral intervention for stress-eating: a comparative pilot study. *Eat Behav* 2014; **15:** 694–9.

Crane C, Barnhofer T, Duggan DS, Hepburn S, Fennell MV, Williams JM. Mindfulness-based cognitive therapy and self-discrepancy in recovered depressed patients with a history of depression and suicidality. *Cognit Ther Res* 2008; **32:** 775–87.

Daubenmier J, Kristeller J, Hecht FM, et al. Mindfulness intervention for stress eating to reduce cortisol and abdominal fat among overweight and obese women: an exploratory randomized controlled study. *J Obes* 2011; **2011:** 1–13.

Daubenmier J, Moran PJ, Kristeller J, et al. Effects of a mindfulness‐based weight loss intervention in adults with obesity: a randomized clinical trial. *Obesity* 2016; **24:** 794–804.

Davis JM, Goldberg SB, Anderson MC, Manley AR, Smith SS, Baker TB. Randomized trial on mindfulness training for smokers targeted to a disadvantaged population. *Subst Use Misuse* 2014; **49:** 571–85.

Davis JM, Manley AR, Goldberg SB, Smith SS, Jorenby DE. Randomized trial comparing mindfulness training for smokers to a matched control. *J Subst Abuse Treat* 2014; **47:** 213–21.

Davis JM, Mills DM, Stankevitz KA, Manley AR, Majeskie MR, Smith SS. Pilot randomized trial on mindfulness training for smokers in young adult binge drinkers. *BMC Complement Altern Med* 2013; **13:** 1–10.

Davis MC, Zautra AJ, Wolf LD, Tennen H, Yeung EW. Mindfulness and cognitive–behavioral interventions for chronic pain: Differential effects on daily pain reactivity and stress reactivity. *J Consult Clin Psychol* 2015; **83:** 24–35.

Day MA, Thorn BE, Ward LC, et al. Mindfulness-based cognitive therapy for the treatment of headache pain: a pilot study. *Clin J Pain* 2014; **30:** 152–61.

de Dios MA, Herman DS, Britton WB, Hagerty CE, Anderson BJ, Stein MD. Motivational and mindfulness intervention for young adult female marijuana users. *J Subst Abuse Treat* 2012; **42:** 56–64.

de Jong M, Lazar SW, Hug K, et al. Effects of mindfulness-based cognitive therapy on body awareness in patients with chronic pain and comorbid depression. *Front Psychol* 2016; **7:** 1–13.

Delgado LC, Guerra P, Perakakis P, Vera MN, del Paso GR, Vila J. Treating chronic worry: Psychological and physiological effects of a training programme based on mindfulness. *Behav Res Ther* 2010; **48:** 873–82.

Dimidjian S, Goodman SH, Felder JN, Gallop R, Brown AP, Beck A. Staying well during pregnancy and the postpartum: A pilot randomized trial of mindfulness-based cognitive therapy for the prevention of depressive relapse/recurrence. *J Consult Clin Psychol* 2016; **84:** 134–45.

Eisendrath SJ, Gillung E, Delucchi KL, et al. A randomized controlled trial of mindfulness-based cognitive therapy for treatment-resistant depression. *Psychother Psychosom* 2016; **85:** 99–110.

Esmer G, Blum J, Rulf J, Pier J. Mindfulness-based stress reduction for failed back surgery syndrome: a randomized controlled trial*. J Am Osteopath Assoc* 2010; **110:** 646–52.

Faucher J, Koszycki D, Bradwejn J, Merali Z, Bielajew C. Effects of CBT versus MBSR treatment on social stress reactions in social anxiety disorder. *Mindfulness* 2016; **7:** 514–26.

Fissler M, Winnebeck E, Schroeter T, et al. An Investigation of the Effects of Brief Mindfulness Training on Self-Reported Interoceptive Awareness, the Ability to Decenter, and Their Role in the Reduction of Depressive Symptoms. *Mindfulness* 2016; **7:** 1170–81.

Fleer J, Schroevers M, Panjer V, Geerts E, Meesters Y. Mindfulness-based cognitive therapy for seasonal affective disorder: a pilot study. *J Affect Disorders* 2014; **168:** 205–9.

Fogarty FA, Booth RJ, Gamble GD, Dalbeth N, Consedine NS. The effect of mindfulness-based stress reduction on disease activity in people with rheumatoid arthritis: a randomised controlled trial. *Ann Rheum Dis* 2015; **74:** 472–4.

Garland EL, Gaylord SA, Boettiger CA, Howard MO. Mindfulness training modifies cognitive, affective, and physiological mechanisms implicated in alcohol dependence: results of a randomized controlled pilot trial. *J Psychoactive Drugs* 2010; **42:** 177–92.

Garland EL, Howard MO. Mindfulness-oriented recovery enhancement reduces pain attentional bias in chronic pain patients. *Psychother Psychosom* 2013; **82:** 311–8.

Garland EL, Manusov EG, Froeliger B, Kelly A, Williams JM, Howard MO. Mindfulness-oriented recovery enhancement for chronic pain and prescription opioid misuse: results from an early-stage randomized controlled trial. *J Consult Clin Psychol* 2014; **82:** 448–59.

Garland EL, Roberts-Lewis A, Tronnier CD, Graves R, Kelley K. Mindfulness-Oriented Recovery Enhancement versus CBT for co-occurring substance dependence, traumatic stress, and psychiatric disorders: Proximal outcomes from a pragmatic randomized trial. *Behav Res Ther* 2016; **77:** 7–16.

Garland EL, Thomas E, Howard MO. Mindfulness-oriented recovery enhancement ameliorates the impact of pain on self-reported psychological and physical function among opioid-using chronic pain patients. *J Pain Symptom Manage* 2014; **48:** 1091–9.

Garland SN, Carlson LE, Stephens AJ, Antle MC, Samuels C, Campbell TS. Mindfulness-based stress reduction compared with cognitive behavioral therapy for the treatment of insomnia comorbid with cancer: a randomized, partially blinded, noninferiority trial. *J Clin Oncol* 2014; **32:** 449–57.

Geschwind N, Peeters F, Drukker M, van Os J, Wichers M. Mindfulness training increases momentary positive emotions and reward experience in adults vulnerable to depression: a randomized controlled trial. *J Consult Clin Psychol* 2011; **79:** 618–28.

Glasner S, Mooney LJ, Ang A, et al. Mindfulness-based relapse prevention for stimulant dependent adults: a pilot randomized clinical trial. *Mindfulness* 2016: DOI 10.1007/s12671-016-0586-9

Godfrin KA, Van Heeringen C. The effects of mindfulness-based cognitive therapy on recurrence of depressive episodes, mental health and quality of life: A randomized controlled study. *Behav Res Ther* 2010; **48:** 738–46.

Goldin P, Ziv M, Jazaieri H, Gross J. Randomized controlled trial of mindfulness-based stress reduction versus aerobic exercise: effects on the self-referential brain network in social anxiety disorder. *Front Hum Neurosci* 2012; **6:** 1–16.

Goldin P, Ziv M, Jazaieri H, Hahn K, Gross JJ. MBSR vs aerobic exercise in social anxiety: fMRI of emotion regulation of negative self-beliefs. *Soc Cogn Affect Neurosci* 2013; **8:** 65–72.

Goldin PR, Morrison A, Jazaieri H, Brozovich F, Heimberg R, Gross JJ. Group CBT versus MBSR for social anxiety disorder: A randomized controlled trial. *J Consult Clin Psychol* 2016; **84:** 427–37.

Greenberg J, Shapero BG, Mischoulon D, Lazar SW. Mindfulness-based cognitive therapy for depressed individuals improves suppression of irrelevant mental-sets. *Eur Arch Psychiatry Clin Neurosci* 2016: DOI 10.1007/s00406-016-0746-x

Gross CR, Kreitzer MJ, Reilly-Spong M, et al. Mindfulness-based stress reduction versus pharmacotherapy for chronic primary insomnia: a randomized controlled clinical trial. *Explore* 2011; **7:** 76–87.

Grossman P, Deuring G, Walach H, Schwarzer B, Schmidt S. Mindfulness-based intervention does not influence cardiac autonomic control or the pattern of physical activity in fibromyalgia during daily life: an ambulatory, multimeasure randomized controlled trial. *Clin J Pain* 2017; **33:** 385–94.

Hanstede M, Gidron Y, Nyklícek I. The effects of a mindfulness intervention on obsessive-compulsive symptoms in a non-clinical student population. *J Nerv Ment Dis* 2008; **196:** 776–9.

Helmes E, Ward BG. Mindfulness-based cognitive therapy for anxiety symptoms in older adults in residential care. *Aging Ment Health* 2017; **21:** 272–8.

Hepark S, Janssen L, de Vries A, et al. The efficacy of adapted MBCT on core symptoms and executive functioning in adults with ADHD: a preliminary randomized controlled trial. *J Atten Disord* 2015: 1–12.

Hepburn SR, Crane C, Barnhofer T, Duggan DS, Fennell MJ, Williams JM. Mindfulness-based cognitive therapy may reduce thought suppression in previously suicidal participants: Findings from a preliminary study. *Br J Clin Psychol* 2009; **48:** 209–15.

Hoge EA, Bui E, Marques L, Metcalf CA, et al. Randomized controlled trial of mindfulness meditation for generalized anxiety disorder: Effects on anxiety and stress reactivity. *J Clin Psychiatry* 2013; **74:** 786–792.

Hoge EA, Bui E, Goetter E, et al. Change in decentering mediates improvement in anxiety in mindfulness-based stress reduction for generalized anxiety disorder. *Cognit Ther Res* 2015; **39:** 228–35.

Huijbers MJ, Spinhoven P, Spijker J, et al. Adding mindfulness-based cognitive therapy to maintenance antidepressant medication for prevention of relapse/recurrence in major depressive disorder: randomised controlled trial. *J Affect Disorders* 2015; **187:** 54–61.

Imani S, Vahid MK, Gharraee B, Noroozi A, Habibi M, Bowen S. Effectiveness of Mindfulness-Based Group Therapy Compared to the Usual Opioid Dependence Treatment. *Iran J Psychiatry* 2015; **10:** 175–84.

Iranshahri B, Jenaabadi H. The Effectiveness of Mindfulness Therapy in Controlling under Treatment Addicts’ Drug Cravings. *Open J Med Psychol* 2015; **4:** 88–98.

Jay K, Brandt M, Hansen K, et al. Effect of individually tailored biopsychosocial workplace interventions on chronic musculoskeletal pain and stress among laboratory technicians: randomized controlled trial. *Pain Physician* 2015; **18:** 459–71.

Jay K, Brandt M, Jakobsen et al. Ten weeks of physical-cognitive-mindfulness training reduces fear-avoidance beliefs about work-related activity: randomized controlled trial. *Medicine* 2016; **95:** e3945.

Jazaieri H, Goldin PR, Werner K, Ziv M, Gross JJ. A randomized trial of MBSR versus aerobic exercise for social anxiety disorder. *J Clin Psychol* 2012; **68:** 715–31.

Jazaieri H, Lee IA, Goldin PR, Gross JJ. Pre-treatment social anxiety severity moderates the impact of mindfulness-based stress reduction and aerobic exercise. *Psychol Psychother T* 2016; **89:** 229–34.

Johannsen M, O’Connor M, O’Toole MS, Jensen AB, Højris I, Zachariae R. Efficacy of mindfulness-based cognitive therapy on late post-treatment pain in women treated for primary breast cancer: A randomized controlled trial. *J Clin Oncol* 2016; **34:** 3390–9.

Kanter G, Komesu YM, Qaedan F, et al. Mindfulness-based stress reduction as a novel treatment for interstitial cystitis/bladder pain syndrome: a randomized controlled trial *Int Urogynecol J* 2016; **27:** 1705–11.

Kaviani H, Hatami N, Javaheri F. The impact of mindfulness-based cognitive therapy (MBCT) on mental health and quality of life in a sub-clinically depressed population. *Arch Psychiatry Psychother* 2012; **1:** 21–8.

Kearney DJ, McDermott K, Malte C, Martinez M, Simpson TL. Effects of participation in a mindfulness program for veterans with posttraumatic stress disorder: a randomized controlled pilot study. *J Clin Psychol* 2013; **69:** 14–27.

Kearns NP, Shawyer F, Brooker JE, et al. Does rumination mediate the relationship between mindfulness and depressive relapse?. *Psychol Psychother T* 2016; **89:** 33–49.

Keune PM, Bostanov V, Hautzinger M, Kotchoubey B. Mindfulness-based cognitive therapy (MBCT), cognitive style, and the temporal dynamics of frontal EEG alpha asymmetry in recurrently depressed patients. *Biol Psychol* 2011; **88:** 243–52.

King AP, Block SR, Sripada RK, et al. Altered default mode network (DMN) resting state functional connectivity following a mindfulness-based exposure therapy for posttraumatic stress disorder (PTSD) in combat veterans of Afghanistan and Iraq. *Depress Anxiety* 2016; **33:** 289–99.

Kocovski NL, Fleming JE, Hawley LL, Huta V, Antony MM. Mindfulness and acceptance-based group therapy versus traditional cognitive behavioral group therapy for social anxiety disorder: A randomized controlled trial. *Behav Res Ther* 2013; **51:** 889–98.

Koszycki D, Benger M, Shlik J, Bradwejn J. Randomized trial of a meditation-based stress reduction program and cognitive behavior therapy in generalized social anxiety disorder. *Behav Res Ther* 2007; **45:** 2518–26.

Koszycki D, Thake J, Mavounza C, Daoust JP, Taljaard M, Bradwejn J. Preliminary Investigation of a Mindfulness-Based Intervention for Social Anxiety Disorder That Integrates Compassion Meditation and Mindful Exposure. *J Altern Complement Med* 2016; **22:** 363–74.

Kristeller J, Wolever RQ, Sheets V. Mindfulness-based eating awareness training (MB-EAT) for binge eating: a randomized clinical trial. *Mindfulness* 2014; **5:** 282–97.

Kuyken W, Byford S, Taylor RS, et al. Mindfulness-based cognitive therapy to prevent relapse in recurrent depression. *J Consult Clin Psychol* 2008; **76:** 966–978.

Kuyken W, Watkins E, Holden E, et al. How does mindfulness-based cognitive therapy work?. *Behav Res Ther* 2010; **48:**1105–12.

Kuyken W, Hayes R, Barrett B, et al. Effectiveness and cost-effectiveness of mindfulness-based cognitive therapy compared with maintenance antidepressant treatment in the prevention of depressive relapse or recurrence (PREVENT): a randomised controlled trial. *Lancet* 2015; **386:** 63–73.

la Cour P, Petersen M. Effects of mindfulness meditation on chronic pain: a randomized controlled trial. *Pain Med* 2015; **16:** 641–52.

Langer ÁI, Cangas AJ, Gallego J. Mindfulness-based intervention on distressing hallucination-like experiences in a nonclinical sample. *Behav Change* 2010; **27:** 176–83.

Langer ÁI, Cangas AJ, Salcedo E, Fuentes B. Applying mindfulness therapy in a group of psychotic individuals: a controlled study. *Behav Cogn Psychother* 2012; **40:** 105–9.

Lee KH, Bowen S, An-Fu B. Psychosocial outcomes of mindfulness-based relapse prevention in incarcerated substance abusers in Taiwan: A preliminary study. *J Subst Use* 2011; **16:** 476–83.

Lipschitz DL, Kuhn R, Kinney AY, Donaldson GW, Nakamura Y. Reduction in salivary α-amylase levels following a mind–body intervention in cancer survivors: an exploratory study. *Psychoneuroendocrino* 2013; **38:** 1521–31.

López-Navarro E, Del Canto C, Belber M, et al. Mindfulness improves psychological quality of life in community-based patients with severe mental health problems: a pilot randomized clinical trial. *Schizophr Res* 2015; **168:** 530–6.

Ma SH, Teasdale JD. Mindfulness-based cognitive therapy for depression: replication and exploration of differential relapse prevention effects. *J Consult Clin Psychol* 2004; **72:** 31–40.

Madani NA, Kananifar N, Atashpour SH, Habil MB. The effects of mindfulness group training on the rate of obsessive-compulsive disorder symptoms on the women in Isfahan City (Iran). *Int Med J* 2013; **20:** 13–7.

Majid SA, Seghatoleslam T, Homan HA, Akhvast A, Habil H. Effect of mindfulness based stress management on reduction of generalized anxiety disorder. *Iran J Pub Health* 2012; **41:** 24–28.

Manicavasgar V, Parker G, Perich T. Mindfulness-based cognitive therapy vs cognitive behaviour therapy as a treatment for non-melancholic depression. *J Affect Disorders* 2011; **130:** 138–44.

Manicavasagar V, Perich T, Parker G. Cognitive predictors of change in cognitive behaviour therapy and mindfulness-based cognitive therapy for depression. *Behav Cogn Psychother* 2012; **40:** 227–32.

Mann J, Kuyken W, O’Mahen H, Ukoumunne OC, Evans A, Ford T. Manual development and pilot randomised controlled trial of mindfulness-based cognitive therapy versus usual care for parents with a history of depression. *Mindfulness* 2016; **7:** 1024–33.

Mason AE, Epel ES, Kristeller J, et al. Effects of a mindfulness-based intervention on mindful eating, sweets consumption, and fasting glucose levels in obese adults: data from the SHINE randomized controlled trial. *J Behav Med* 2016; **39:** 201–13.

Mason AE, Epel ES, Aschbacher K, et al. Reduced reward-driven eating accounts for the impact of a mindfulness-based diet and exercise intervention on weight loss: Data from the SHINE randomized controlled trial. *Appetite* 2016; **100:** 86–93.

McIndoo CC, File AA, Preddy T, Clark CG, Hopko DR. Mindfulness-based therapy and behavioral activation: A randomized controlled trial with depressed college students. *Behav Res Ther* 2016; **77:** 118–28.

Meadows GN, Shawyer F, Enticott JC, et al. Mindfulness-based cognitive therapy for recurrent depression: A translational research study with 2-year follow-up. *Aust N Z J Psychiatry* 2014: 0004867414525841; 1–13.

Michalak J, Schultze M, Heidenreich T, Schramm E. A randomized controlled trial on the efficacy of mindfulness-based cognitive therapy and a group version of cognitive behavioral analysis system of psychotherapy for chronically depressed patients. *J Consult Clin Psychol* 2015; **83:** 951–63.

Michalak J, Probst T, Heidenreich T, Bissantz N, Schramm E. Mindfulness-based cognitive therapy and a group version of the cognitive behavioral analysis system of psychotherapy for chronic depression: follow-up data of a randomized controlled trial and the moderating role of childhood adversities. Psychother Psychosom 2016; **85:** 378-80.

Miller CK, Kristeller JL, Headings A, Nagaraja H, Miser WF. Comparative effectiveness of a mindful eating intervention to a diabetes self-management intervention among adults with type 2 diabetes: a pilot study. *J Acad Nutr Diet* 2012; **112:** 1835–42.

Miller CK, Kristeller JL, Headings A, Nagaraja H. Comparison of a mindful eating intervention to a diabetes self-management intervention among adults with type 2 diabetes: a randomized controlled trial. *Health Educ Behav* 2014; **41:** 145–54.

Mitchell JT, McIntyre EM, English JS, Dennis MF, Beckham JC, Kollins SH. A pilot trial of mindfulness meditation training for ADHD in adulthood: impact on core symptoms, executive functioning, and emotion dysregulation. *J Atten Disord* 2013: doi: 10.1177/1087054713513328

Moore RC, Depp CA, Wetherell JL, Lenze EJ. Ecological momentary assessment versus standard assessment instruments for measuring mindfulness, depressed mood, and anxiety among older adults. *J Psychiat Res* 2016; **75:** 116–23.

Morone NE, Greco CM, Weiner DK. Mindfulness meditation for the treatment of chronic low back pain in older adults: a randomized controlled pilot study. *Pain* 2008; **134:** 310–9.

Morone NE, Rollman BL, Moore CG, Li Q, Weiner DK. A mind–body program for older adults with chronic low back pain: results of a pilot study. *Pain Med* 2009; **10:** 1395–407.

Morone NE, Greco CM, Moore CG, et al. A mind-body program for older adults with chronic low back pain: a randomized clinical trial. *JAMA Intern Med* 2016; **176:** 329–37.

Nakamura Y, Lipschitz DL, Kuhn R, Kinney AY, Donaldson GW. Investigating efficacy of two brief mind–body intervention programs for managing sleep disturbance in cancer survivors: a pilot randomized controlled trial. *J Cancer Surviv* 2013; **7:** 165–82.

Nassif TH, Chapman JC, Sandbrink F, et al. Mindfulness meditation and chronic pain management in Iraq and Afghanistan veterans with traumatic brain injury: a pilot study. *Mil Behav Health* 2016; **4:** 82–9.

Niles BL, Klunk-Gillis J, Ryngala DJ, Silberbogen AK, Paysnick A, Wolf EJ. Comparing mindfulness and psychoeducation treatments for combat-related PTSD using a telehealth approach. *Psychol Trauma* 2012; **4:** 538–47.

Omidi A, Mohammadkhani P, Mohammadi A, Zargar F. Comparing mindfulness based cognitive therapy and traditional cognitive behavior therapy with treatments as usual on reduction of major depressive disorder symptoms. *Iran Red Crescent Me* 2013; **15:** 142–6.

Ong JC, Manber R, Segal Z, Xia Y, Shapiro S, Wyatt JK. A randomized controlled trial of mindfulness meditation for chronic insomnia. *Sleep* 2014; **37:** 1553–1563B.

Panahi F, Faramarzi M. The effects of mindfulness-based cognitive therapy on depression and anxiety in women with premenstrual syndrome. *Depress Res Treat* 2016; **2016:** 1–7.

Parra-Delgado M, Latorre-Postigo JM. Effectiveness of mindfulness-based cognitive therapy in the treatment of fibromyalgia: a randomised trial. *Cognitive Ther Res* 2013; **37:** 1015–26.

Perich T, Manicavasagar V, Mitchell PB, Ball JR, Hadzi-Pavlovic D. A randomized controlled trial of mindfulness-based cognitive therapy for bipolar disorder. *Acta Psychiat Scand* 2013; **127:** 333–43.

Piet J, Hougaard E, Hecksher MS, Rosenberg NK. A randomized pilot study of mindfulness-based cognitive therapy and group cognitive-behavioral therapy for young adults with social phobia. *Scand J Psychol* 2010; **51:** 403–10.

Plews-Ogan M, Owens JE, Goodman M, Wolfe P, Schorling J. A pilot study evaluating mindfulness-based stress reduction and massage for the management of chronic pain. *J Gen Intern Med* 2005; **20:** 1136–8.

Polusny MA, Erbes CR, Thuras P, et al. Mindfulness-based stress reduction for posttraumatic stress disorder among veterans: a randomized clinical trial. *JAMA* 2015; **314:** 456–65.

Possemato K, Bergen-Cico D, Treatman S, Allen C, Wade M, Pigeon W. A randomized clinical trial of primary care brief mindfulness training for veterans with PTSD. *J Clin Psychol* 2016; **72:** 179–93.

Pots WT, Meulenbeek PA, Veehof MM, Klungers J, Bohlmeijer ET. The efficacy of mindfulness-based cognitive therapy as a public mental health intervention for adults with mild to moderate depressive symptomatology: a randomized controlled trial. *PLoS One* 2014; **9:** e109789.

Pradhan EK, Baumgarten M, Langenberg P, et al. Effect of mindfulness-based stress reduction in rheumatoid arthritis patients. *Arthrit Care Res* 2007; **57:** 1134–42.

Rungreangkulkij S, Wongtakee W, Thongyot S. Buddhist group therapy for diabetes patients with depressive symptoms. *Arch Psychiat Nurs* 2011; **25:** 195–205.

Schmidt S, Grossman P, Schwarzer B, Jena S, Naumann J, Walach H. Treating fibromyalgia with mindfulness-based stress reduction: results from a 3-armed randomized controlled trial. *Pain* 2011; **152:** 361–9.

Schoenberg PL, Hepark S, Kan CC, Barendregt HP, Buitelaar JK, Speckens AE. Effects of mindfulness-based cognitive therapy on neurophysiological correlates of performance monitoring in adult attention-deficit/hyperactivity disorder. *Clin Neurophysiol* 2014; **125:** 1407–16.

Schuver KJ, Lewis BA. Mindfulness-based yoga intervention for women with depression. *Complement Ther Med* 2016; **26:** 85–91.

Segal ZV, Bieling P, Young T, et al. Antidepressant monotherapy vs sequential pharmacotherapy and mindfulness-based cognitive therapy, or placebo, for relapse prophylaxis in recurrent depression. *Arch Gen Psychiat* 2010; **67:** 1256–64.

Shahar B, Britton WB, Sbarra DA, Figueredo AJ, Bootzin RR. Mechanisms of change in mindfulness-based cognitive therapy for depression: Preliminary evidence from a randomized controlled trial. *Int J Cogn Ther* 2010; **3:** 402–18.

Shallcross AJ, Gross JJ, Visvanathan PD, et al. Relapse prevention in major depressive disorder: Mindfulness-based cognitive therapy versus an active control condition. *J Consult Clin Psychol* 2015; **83:** 964–75.

Singh NN, Lancioni GE, Myers RE, Karazsia BT, Winton AS, Singh J. A randomized controlled trial of a mindfulness-based smoking cessation program for individuals with mild intellectual disability. *Int J Ment Health Ad* 2014; **12:** 153–68.

Strauss C, Hayward M, Chadwick P. Group person-based cognitive therapy for chronic depression: a pilot randomized controlled trial. *Brit J Clin Psychol* 2012; **51:** 345–50.

Tang YY, Tang R, Posner MI. Brief meditation training induces smoking reduction. *P Natl Acad Sci USA* 2013; **110:** 13971–5.

Teasdale JD, Segal ZV, Williams JM, Ridgeway VA, Soulsby JM, Lau MA. Prevention of relapse/recurrence in major depression by mindfulness-based cognitive therapy. *J Consult Clin Psychol* 2000; **68:** 615–623.

Thompson NJ, Walker ER, Obolensky N, et al. Distance delivery of mindfulness-based cognitive therapy for depression: project UPLIFT. *Epilepsy Behav* 2010; **19:** 247–54.

Tovote KA, Fleer J, Snippe E, et al. Individual mindfulness-based cognitive therapy and cognitive behavior therapy for treating depressive symptoms in patients with diabetes: results of a randomized controlled trial. *Diabetes Care* 2014; **37:** 2427–34.

Turner JA, Anderson ML, Balderson BH, Cook AJ, Sherman KJ, Cherkin DC. Mindfulness-based stress reduction and cognitive behavioral therapy for chronic low back pain: similar effects on mindfulness, catastrophizing, self-efficacy, and acceptance in a randomized controlled trial. *Pain* 2016; **157:** 2434–44.

Van Aalderen JR, Donders AR, Giommi F, Spinhoven P, Barendregt HP, Speckens AE. The efficacy of mindfulness-based cognitive therapy in recurrent depressed patients with and without a current depressive episode: a randomized controlled trial. *Psychol Med* 2012; **42:** 989–1001.

Vidrine JI, Spears CA, Heppner WL, et al. Efficacy of mindfulness-based addiction treatment (MBAT) for smoking cessation and lapse recovery: A randomized clinical trial. *J Consult Clin Psychol* 2016; **84:** 824–838.

Vøllestad J, Sivertsen B, Nielsen GH. Mindfulness-based stress reduction for patients with anxiety disorders: Evaluation in a randomized controlled trial. *Behav Res Therapy* 2011; **49:** 281–8.

Wang LQ, Chien WT, Yip LK, Karatzias T. A randomized controlled trial of a mindfulness-based intervention program for people with schizophrenia: 6-month follow-up. *Neuropsych Dis Treat* 2016; **12:** 3097–3110.

Wells RE, Burch R, Paulsen RH, Wayne PM, Houle TT, Loder E. Meditation for migraines: a pilot randomized controlled trial. *Headache* 2014; **54:** 1484–95.

Williams JM, Teasdale JD, Segal ZV, Soulsby J. Mindfulness-based cognitive therapy reduces overgeneral autobiographical memory in formerly depressed patients. *J Abnorm Psych* 2000; **109:** 150–5.

Williams JM, Alatiq Y, Crane C, et al. Mindfulness-based cognitive therapy (MBCT) in bipolar disorder: Preliminary evaluation of immediate effects on between-episode functioning. *J Affect Disorders* 2008; **107:** 275–9.

Williams JM, Crane C, Barnhofer T, et al. Mindfulness-based cognitive therapy for preventing relapse in recurrent depression: a randomized dismantling trial. *J Consult Clin Psychol* 2014; **82:** 275–86.

Witkiewitz K, Greenfield BL, Bowen S. Mindfulness-based relapse prevention with racial and ethnic minority women. *Addict Behav* 2013; **38:** 2821–4.

Witkiewitz K, Warner K, Sully B, et al. Randomized trial comparing mindfulness-based relapse prevention with relapse prevention for women offenders at a residential addiction treatment center. *Subst Use Misuse* 2014; **49:** 536-46.

Wong SY. Effect of mindfulness-based stress reduction programme on pain and quality of life in chronic pain patients: a randomised controlled clinical trial. *Hong Kong Med J* 2009; **15:** 13–4.

Wong SY, Chan FW, Wong RL, et al. Comparing the effectiveness of mindfulness-based stress reduction and multidisciplinary intervention programs for chronic pain: a randomized comparative trial. *Clin J Pain* 2011; **27:** 724–34.

Wong MY, Ree MJ, Lee CW. Enhancing CBT for chronic insomnia: A randomised clinical trial of additive components of mindfulness or cognitive therapy. *Clin Psychol Psychother T* 2015; doi: 10.1002/cpp.1980.

Wong SY, Yip BH, Mak WW, et al. Mindfulness-based cognitive therapy v. group psychoeducation for people with generalised anxiety disorder: Randomised controlled trial. *Brit J Psychiat* 2016; **209:** 68–75.

Zangi HA, Mowinckel P, Finset A, et al. A mindfulness-based group intervention to reduce psychological distress and fatigue in patients with inflammatory rheumatic joint diseases: A randomised controlled trial. *Ann Rheum Dis* 2012; **71:** 911–7.

Zautra AJ, Davis MC, Reich JW, et al. Comparison of cognitive behavioral and mindfulness meditation interventions on adaptation to rheumatoid arthritis for patients with and without history of recurrent depression. *J Consult Clin Psychol* 2008; **76:** 408–21.

Zemestani M, Ottaviani C. Effectiveness of mindfulness-based relapse prevention for co-occurring substance use and depression disorders. *Mindfulness* 2016; **7:** 1347–55.

Zgierska AE, Burzinski CA, Cox J, et al. Mindfulness meditation and cognitive behavioral therapy intervention reduces pain severity and sensitivity in opioid-treated chronic low back pain: pilot findings from a randomized controlled trial. *Pain Med* 2016; **17:** 1865–81.

Zhang JX, Liu XH, Xie XH, et al. Mindfulness-based stress reduction for chronic insomnia in adults older than 75 years: a randomized, controlled, single-blind clinical trial. *Explore* 2015; **11:** 180–5.
